# Chagasic cardiomyopathy is marked by a unique signature of activated CD4^+^ T cells

**DOI:** 10.1186/s12967-022-03761-5

**Published:** 2022-11-30

**Authors:** Gregório Guilherme Almeida, Inga Rimkute, Isabela Natália Pascoal Campos do Vale, Thomas Liechti, Priscilla Miranda Henriques, Ester Roffe, Fernanda Fortes de Araújo, Manoel Otávio da Costa Rocha, Silvana Maria Elói Santos, Olindo Assis Martins-Filho, Dragana Jankovic, Alan Sher, Andrea Teixeira-Carvalho, Mario Roederer, Lis Ribeiro do Valle Antonelli

**Affiliations:** 1 Laboratório de Biologia e Imunologia de Doenças Infecciosas e Parasitárias, Instituto René Rachou, Fundação Oswaldo Cruz-FIOCRUZ, Minas Gerais Belo Horizonte, Brazil; 2 Vaccine Research Center, National Institute of Allergy and Infectious Diseases, National Institutes of Health, Bethesda, MD USA; 3 Laboratory of Molecular Immunology, Molecular Signaling Section, National Institutes of Allergy and Infectious Diseases, National Institutes of Health, Bethesda, MD USA; 4 Grupo Integrado de Pesquisas em Biomarcadores, Instituto René Rachou, Fundação Oswaldo Cruz-FIOCRUZ, Belo Horizonte, Minas Gerais Brasil; 5 Departamento de Clínica Médica, Curso de Pós-Graduação em Infectologia e Medicina Tropical, Universidade Federal de Minas Gerais, Belo Horizonte, Minas Gerais Brazil; 6 Departamento de Propedêutica Complementar, Faculdade de Medicina, Universidade Federal de Minas Gerais, Belo Horizonte, Brazil; 7 Immunobiology Section, Laboratory of Parasitic Diseases, National Institute of Allergy and Infectious Diseases, National Institutes of Health, Bethesda, MD USA

**Keywords:** Chagas disease, *Trypanosoma cruzi*, Cardiomyopathy, CD4^+^ T cells, Multifunctional, Cytotoxic, Regulatory, Biomarkers

## Abstract

Chagas disease is a neglected tropical disease in Latin America and an imported emerging disease worldwide. Chronic Chagas disease cardiomyopathy (CCC) is the most prominent clinical form and can lead to heart failure, thromboembolism, and sudden death. While previous reports have supported a role for CD4^+^ T lymphocytes in the pathogenesis of CCC a comprehensive analysis of these cells during different clinical forms is lacking. Here, we used high-dimensional flow cytometry to assess the diversity of circulating CD4^+^ T cells in patients with distinct clinical forms. We found increased frequencies of CD4^+^CD69^+^ T cells in patients compared to controls. CD39^+^ regulatory T cells, represented by mesocluster 6 were reduced in mild CCC patients compared to controls. Cytotoxic CD4^+^ T cells co-expressing granzyme B and perforin were expanded in patients with Chagas disease and were higher in patients with mild CCC compared to controls. Furthermore, patients with mild CCC displayed higher frequencies of multifunctional effector memory CD4^+^ T cells. Our results demonstrate an expansion in activated CD4^+^ T cells and a decrease in a functional subset of regulatory T cells associated with the onset of Chagas cardiomyopathy, suggesting their role in the establishment of cardiac lesions and as potential biomarkers for disease aggravation.

**Supplementary Information:**

The online version contains supplementary material available at 10.1186/s12967-022-03761-5.

## Background

Chagas disease is an anthroponosis caused by the protozoa parasite *Trypanosoma cruzi* and is a leading cause of chronic cardiomyopathy in Latin America [1]. In its natural condition, the disease is transmitted by infected feces from triatomines. Recently, contaminated food has emerged as an important transmission source, and, to a minor extent, infections happen by congenital transmission and blood transfusion [1, 2]. The disease has spread from its original boundaries in South America through international migration and has become a new concern for blood donation worldwide [3].

About 30% of the infection progresses to cardiomyopathy and 8–10% progress to mega syndromes, mainly in esophagus and colon, culminating in organ hypertrophy and dilatation [1]. Chronic Chagas disease cardiomyopathy (CCC) is a life-threatening disease that leads to direct complications in the cardiovascular system, such as conduction disorders and heart failure, being the most important cause of death in infected individuals [4]. CCC is characterized by a multi-focal inflammatory infiltrate within the myocardium, mainly characterized by infiltrating CD8^+^ and CD4^+^ T cells, macrophages, and progressive interstitial fibrosis [5, 6]. The disease’s progression is highly variable, and individuals may stay in an indeterminate clinical form for decades until the first clinical symptoms emerge. Chronic inflammation results in a tissue remodeling that leads to loss of function, and in several cases to the need for heart transplantation [7].

The mechanisms underlying the progression of CCC are poorly understood and probably multifactorial. The persistence of *T. cruzi* in tissue provides the antigenic load that sustains local inflammation, although the presence of the parasite in myocardium is scarce [5, 8, 9]. CD8^+^ T cells are essential for parasite control in murine models by promoting cell death of infected cardiomyocytes through main-histocompatibility complex class-1 (MHC-I) antigen presentation [10]. In contrast, the role of CD4^+^ T cells has been more obscure in the pathogenesis of CCC. *Trypanosoma cruzi*-specific IFN-γ^+^CD4^+^ T cells are increased in CCC patients after antigenic recall when compared to patients in the indeterminate form of Chagas disease [11, 12]. In addition, the IFN-γ-induced chemokine receptors CCR5 and CXCR3 are upregulated in CD4^+^ T cells during established cardiomyopathy suggesting the participation of a type-1 T helper-mediated immunity (Th_1_) in the disease progression [13, 14]. Along with the higher proportion of Th_1_ cells in CCC patients, a lower frequency of multifunctional CD4^+^ T cells in the circulation and a higher frequency of senescent and/or exhausted helper T cells have been associated with the progression of the heart disease [15, 16]. Murine models shed light on mechanistic processes in which CD4^+^ T cells may regulate or promote myocarditis [17, 18], but whether the CD4^+^ T cell accumulation in myocardium is a cause or a consequence of progressive cardiomyopathy, is still under investigation.

The complex network of CD4^+^ T cell phenotypes during CCC suggests a shift towards inflammation while immunoregulatory mechanisms seem to be progressively damped [8, 19, 20]. In this sense, the role of specific T helper phenotypes in either promoting or regulating tissue damage deserves further exploration. Also, the lack of reliable markers that correlate with the progression of CCC urges the need for biomarkers that enable the prediction of clinical progression [21].

Here, we used high dimensional flow cytometry to phenotype CD4^+^ T cells from patients in different stages of chronic Chagas disease compared to healthy individuals. We found phenotypic signatures using unsupervised data analysis, which are associated with the immunopathological processes of CCC progression.

## Methods

### Study population

Patients diagnosed with Chagas disease were recruited at Alda Lima Falcão Outpatient Clinic at René Rachou Institute (IRR), Belo Horizonte, Minas Gerais, Brazil. The inclusion criteria were individuals between 18 and 65 years old and with positive serology for Chagas in at least two different diagnostic methods, as established by the II Brazilian Consensus on Chagas disease, 2016 [1]. The exclusion criteria included severe dilated cardiomyopathy, renal and/or liver failure, and comorbidities that significantly shortened life expectancy. The project was approved by the committee of Ethics in Human Research (CAAE: 95998418.8.0000.5091).

A practitioner classified patients into the following three clinical forms [14, 22]: group A (indeterminate)—patients serologically positive for *T. cruzi*, but with undetectable cardiac disease, clinical signs or symptoms; group B1 (mild cardiomyopathy)— patients with structural cardiopathy displaying alteration in the echocardiogram (ECC) and electrocardiogram (ECG), but normal global ventricular function and neither current nor previous signs and symptoms of heart failure; group B2-C-D (established cardiomyopathy)—patients with marked cardiomyopathy with changes ranging from global ventricular dysfunction to symptoms of heart failure. The control group was composed of nine healthy donors from the blood bank of the National Institutes of Health (USA). Additionally, five healthy donors from Brazil were included in some of the supervised analyses. Controls were serologically negative for *T. cruzi*. A brief description of each patient’s metadata and demographics is available in Additional file 5: Table S1.

### PBMC isolation

Peripheral blood was collected in sodium heparin tubes and plasma was removed by centrifugation at 1000×*g* at 10 °C. Cells were diluted 1:1 (v/v) in RPMI 1640 and then peripheral blood mononuclear cells (PBMC) were harvested by centrifugation at 700×*g* for 40 min at 22 °C using Ficoll-Paque gradient (Sigma). PBMC were washed twice in 30 mL of RPMI 1640 and cell concentration and viability were determined by automatic cell counter Countess II FL (Invitrogen Thermo Fisher Scientific). PBMC were frozen in fetal calf serum (FCS, Gibco) with 10% dimethylsulfoxide (DMSO; Sigma) at − 80 °C for 24 h in a Freezing Container (Mr. Frosty™, Thermo Scientific) and then maintained in liquid nitrogen until use.

### Soluble *T. cruzi* antigen (STcA)

*Trypanosoma cruzi* Y strain soluble antigen (STcA) was obtained from trypomastigotes sonicated by six cycles of 40 W for 1 min, alternating with 30 s on ice. Parasite lysate was analyzed under an optical microscope to confirm the total disruption of the parasites and the material was subjected to centrifugation at 30,000×*g*, 60 min at 4 °C. The supernatant was collected, dialyzed in PBS for 48 h at 4 °C, filtered through a 0.45 μm filter, and kept in 1 mL aliquots at − 80 °C. The protein concentration was obtained by the Bradford method and inspected in a 12% PAGE to confirm protein integrity and characteristic bands.

### Cell culture and antigenic recall

Cryopreserved PBMC were thawed using the “thawsome” tube adaptor [23] on a 15mL tube containing 10 mL RPMI with 25 U/mL of benzonase nuclease. PBMC (2 × 10^6^) were stimulated with 10 µg/mL of STcA for 7 h (37 °C, 5% CO_2_). Anti-CD107a (Alexa Fluor 700, clone H4A3) was added to the cultures (1:158 v/v) to reveal lytic vesicles that are transiently located on the cell surface upon activation-dependent degranulation [24]. Protein transport inhibitors (Brefeldin and Monensin, 1:2000 v/v) were then added and cells were incubated for an additional 6 h before the staining protocol.

### Cell staining for flow cytometry

High-dimensional flow cytometry was performed based on the panel previously described with modifications [24]. PBMC were washed with PBS and then stained for 15 min with viability dye (Live/dead UV blue, Thermo). Cells were washed with staining buffer (RPMI with 4% heat-inactivated newborn calf serum) and stained for 30 min with monoclonal antibodies against extracellular antigens: CD28 (PE, clone CD28.2), TCRγδ (PE-Cy5, clone B1), CD3 (APC-H7, clone SK7), CCR7 (BUV395, clone 150503), CD25 (BUV563, clone 2A3), CD39 (BUV661, clone TU66), CD95 (BUV737, clone DX27), CD8 (BUV 805, clone SK1), CD45RO (BV570, clone UCHL1) and CD27 (BV786, clone L128). Cells were washed twice, fixed and permeabilized for 45 min, 4 °C in the dark according to the manufacturers’ protocol (Foxp3 permeabilization buffer, ThermoFisher). Intracellular staining was performed for 30 min using monoclonal antibodies against the following antigens: granzyme B (FITC, clone GB11), T-bet (BB630, clone 4B10), IL-13 (BB660, clone JES10-5A2), IFN-γ (BB700, clone B27), RORγt (BB790, clone Q21-559), perforin (Alexa fluor 594, clone B-D48), FoxP3 (Pe-Cy5.5, clone PCH101), IL-22 (PE-Cy7, clone 22URTI), IL-21 (Alexa fluor 647, clone 3A3-N2.1), CD4 (BUV496, clone SK3), IL-2 (BV421, clone MQ1-17H12), CD154 (BV480, clone TRAP1), IL-17 A (BV605, clone BL168), Ki67 (BV650, clone B56), CD69 (BV711, clone FN50), TNF (BV750, clone Mab11). After washed, cells were suspended in staining buffer and acquired in a FACSymphony A5 at the Vaccine Research Center/NIH cytometer facilities. Details on the antibodies, fluorochromes and clones are available in Additional file 6: Table S2.

### Statistical analysis

Cell frequencies defined manually (supervised) or by the unsupervised approach were calculated on the parental population or on total CD4^+^ T cells, respectively. Cell frequency and mean fluorescence intensity values were extracted in FlowJo (v10). For unsupervised analysis we used packages FlowSOM(v2.1.10), Pheatmap (v1.0.12) and Rtsne (v0.15) in R (v4.1.2). We used the package FlowSOM for unsupervised clustering [25]. For clustering analysis we determined 100 clusters and 50 metaclusters and their distribution was represented in minimum spanning trees (MST). Clusters were further manually organized in seven mesoclusters (MC) based on similar expression pattern and location on MST. Heatmaps were generated with package Pheatmap with hierarchical clustering calculated by using Manhattan distance. Dimensionality reduction was performed using tSNE (t-distributed Stochastic Neighbor Embedding) to visualize high-dimensional data, with modifications: tSNE was set to run 5000 iterations with a learning rate (eta) of 10,000 and an exaggeration factor set to 36 until 1000 iterations, after which momentum was set to begin. Cluster frequencies were exported in csv format files for statistical analysis. Frequencies from each cell population, separated in the clinical groups, were analyzed using the GraphPad Prism (v.8). The Shapiro-Wilk test was used to assess whether the variables in each clinical group had a parametric distribution. For comparisons between clinical groups, the Kruskal-Wallis test was used followed by the Dunn’s test for multiple comparisons with the family error rate corrected by the Bonferroni method. Data were represented in dot plots over bar plots, considering the median and the interquartile ranges. Significant differences (p < 0.05) were represented with asterisks.

## Results

### Supervised analysis of CD4^+^ T cells reveals the increased effector compartment in patients with Chagas disease

**Fig. 1:**
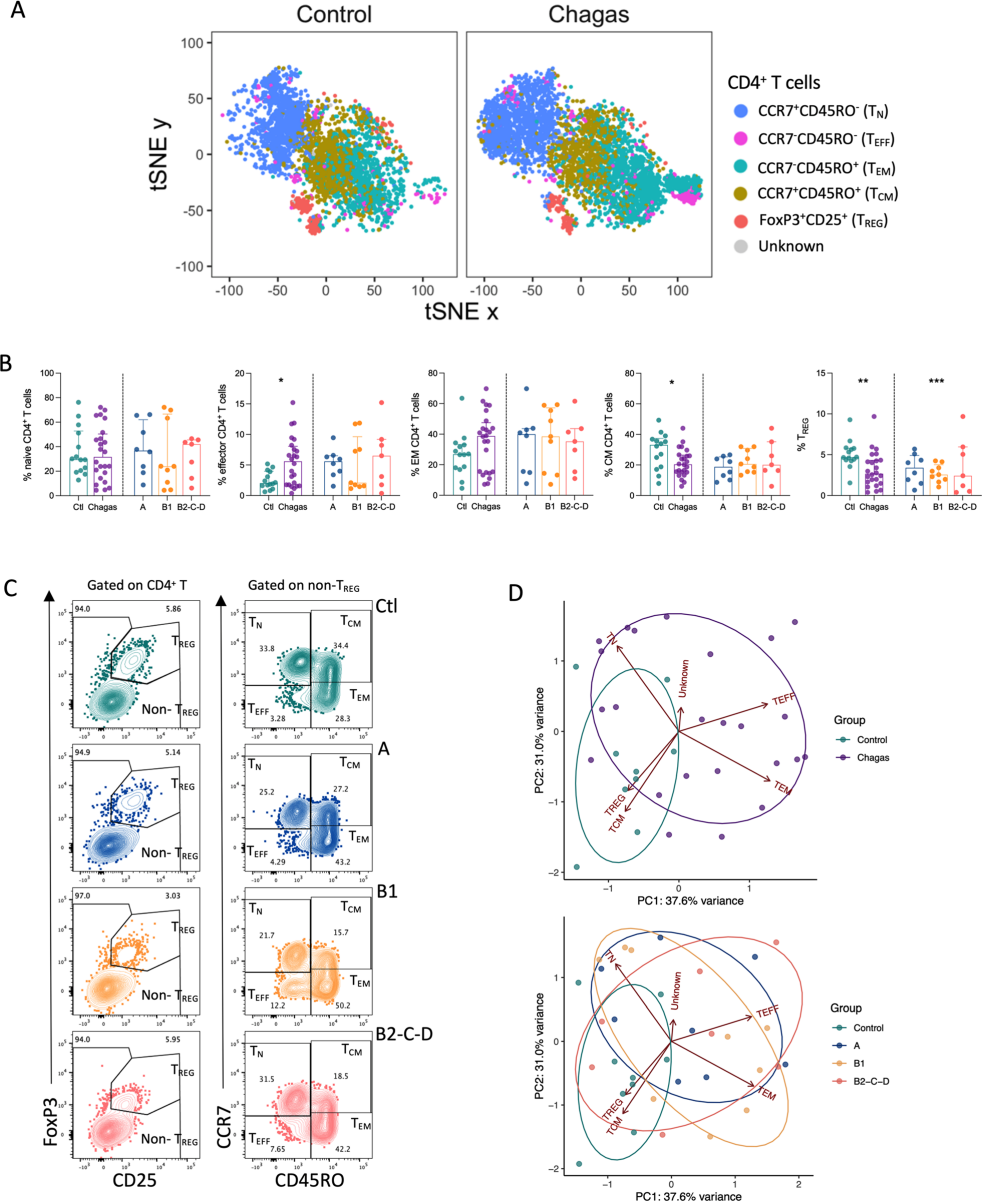
Chagas disease induces increased frequencies of CD4^+^ T cell populations with effector phenotype. **A** tSNE depicts CD4^+^ T cell subpopulations (regulatory T cells, T_REG_, FoxP3^+^CD25^+^; central memory, T_CM_, CCR7^+^CD45RO^+^; effector memory, T_EM_, CCR7^−^CD45RO^+^; effector, T_EFF_, CCR7^−^CD45RO^−^; naïve, T_N_, CCR7^+^CD45RO^−^) from uninfected controls (Ctl, n = 14) and patients with Chagas disease (Chagas, n = 24). Plots are normalized to represent the same number of events. **B** Frequencies of regulatory T cells and memory subsets are shown. Bars represent the median and interquartile range, respectively. Asterisks represent significant differences between the assigned group and controls. *p < 0.05, **p < 0.01, ***p < 0.001. **C** Representative contour plots show the gating strategy for T_REG_ and non-T_REG_ (left panel), and memory CD4^+^ T cells defined by the expression of CCR7 and CD45RO (right panel) from control and patients in different stages of Chagas disease (top to bottom). **D** Principal-component analysis (PCA) for CD4^+^ T cell subpopulations from uninfected controls and infected patients in different stages of Chagas disease: A (indeterminate, n = 8), B1 (mild cardiomyopathy, n = 9), B2-C-D (established cardiomyopathy, n = 7)

To investigate the CD4^+^ T cell compartment during chronic chagasic infection, we analyzed peripheral blood from patients displaying different forms of Chagas disease using high-dimensional flow cytometry. The CD4^+^ T cell compartment was initially analyzed by a manual gating strategy. We defined regulatory CD4^+^ T cells (T_REG_) based on the co-expression of Foxp3 and CD25. Among non-T_REG_ we assessed the memory populations according to the expression of CCR7 and CD45RO. The frequencies of CCR7^−^ T cells were increased in patients (n = 24), resulting in a significative expansion of effector T cells (T_EFF_, CCR7^−^CD45RO^−^CD4^+^) compared to healthy controls (n = 14) (Fig. 1A and B). In contrast, lower frequencies in central memory T cells (T_CM_, CCR7^+^CD45RO^+^CD4^+^) and T_REG_ were observed in patients compared to controls (Fig. 1A and B). Except for T_REG_ cells, which were lower in group B1 (n = 9) compared to controls, no differences were observed within the frequencies of any compartment analyzed among clinical groups: indeterminate (A), mild (B1) and established cardiomyopathy (B2-C-D) (Fig. 1A and B). No differences were observed in the frequencies of naïve (T_N_, CCR7^+^CD45RO^−^CD4^+^) or effector memory population (T_EM_, CCR7^−^CD45RO^+^CD4^+^) among clinical groups (Additional file 1: Fig. S1 A).

Principal component analyses (PCA) were performed using the frequencies of manually gated populations (Fig. 1C) to identify phenotype signatures that may segregate a specific clinical form. PCA suggests a shift towards an expanded compartment of T_EFF_ and T_EM_ during Chagas disease (Fig. 1D, upper panel). However, PCA does not clearly segregate the clinical forms of Chagas disease (Fig. 1D, lower panel). After defining the main CD4^+^ T cell compartments, we explored the expression of the remaining markers among these populations using an unsupervised analysis.

### Unsupervised analysis of CD4^+^ T cells reveals the heterogeneity of memory T cell compartment

**Fig. 2:**
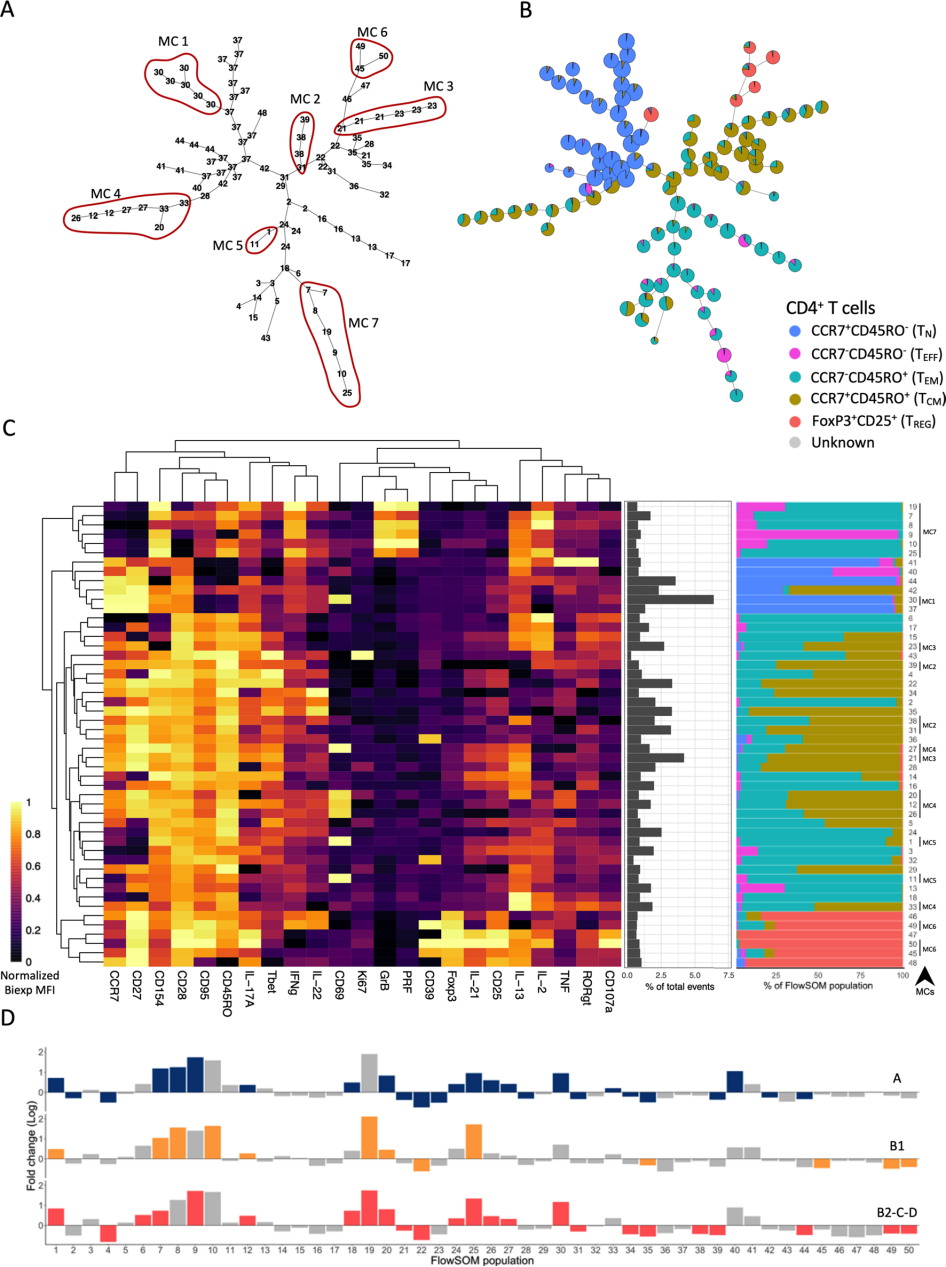
Unsupervised clustering of CD4^+^ T cells reveals unique characteristics of patients with Chagas disease. Minimum spanning trees (MST) show 100 clusters representing the numbers of each of the 50 FlowSOM populations (**A**) and their composition as defined by manual gating of CD4^+^ T cell subpopulations (T_REG_, T_CM_, T_EM_, T_EFF_, T_N_) (**B**). **C** Heatmap depicts the expression of 23 molecules, scaled by each marker, across the 50 FlowSOM populations. Proportion (middle panel) and composition (right panel) of each FlowSOM population within CD4^+^ T cells. FlowSOM populations were clustered based on hierarchical clustering by similar expression patterns and were further grouped in mesoclusters (MC1-7). These mesoclusters are highlighted in the MST (**A**). **D** Fold changes of FlowSOM population frequencies from infected patients in different stages of Chagas disease over controls. Colored bars highlight FlowSOM populations with the statistical difference between patients with clinical forms of Chagas disease (blue, A; orange, B1; coral, B2-C-D) and controls

To further explore potential CD4^+^ T cell immunophenotypes, which were not captured by our manual analysis, we have performed an unsupervised clustering using the Self-Organizing Maps algorithm (FlowSOM) [25]. The T_N_ population was topologically grouped in a specific branch of the MST (Fig. 2A, B) and most of these clusters were highly pure with a minor contamination of T_CM_ and T_EM_ (Fig. 2B, C right panel, blue). T_REG_ were distributed along five clusters located in a terminal branch emerging from T_CM_ and one cluster emerging from T_N_ cells (Fig. 2B, C right panel, salmon). These clusters were composed mainly of T_REG_ with a minor contamination of T_EM_ and T_CM_. Clusters mainly composed of T_CM_ also had significant proportions of T_EM_ and were distributed along two branches near to T_N_ and T_REG_ (Fig. 2B, C right panel, brown). Clusters mainly composed of T_EM_ were grouped in one branch with most of the clusters with a minor proportion of T_EFF_ and T_CM_ (Fig. 2B, C right panel, turquoise). T_EFF_ were mostly concentrated in cluster 9 but were also represented in clusters topologically located among T_N_ and T_EM_ (Fig. 2A, B, C right panel, pink).

The unsupervised strategy provided an overview of the heterogeneity of different clusters (Fig. 2B), the expression levels of each marker (Fig. 2C, heatmap) and the representation of the manually gated populations on the unsupervised strategy (Fig. 2C, right panel). Among the 50 metaclusters, 32 had different frequencies in patients compared to controls (Additional file 2: Fig. S2). Several metaclusters showed differential frequencies between patients in different clinical forms and controls. For example, the frequency of metacluster 19, mainly composed of T_EM_ and T_EFF_ with an activated phenotype, is increased almost 125-fold in group B1 and 54-fold in B2-C-D compared to controls (Fig. 2D, Additional file 1: Fig. S1B). Based on the similar expression pattern and location on MST, clusters that had similar statistical differences were grouped in seven mesoclusters (Fig. 2A, C setae lower right). Thus, we explored the metaclusters and mesoclusters with differences in frequencies between controls and patients, and among clinical forms.

### CD69 expression is a hallmark of chronic cardiac disease in Chagas patients

**Fig. 3:**
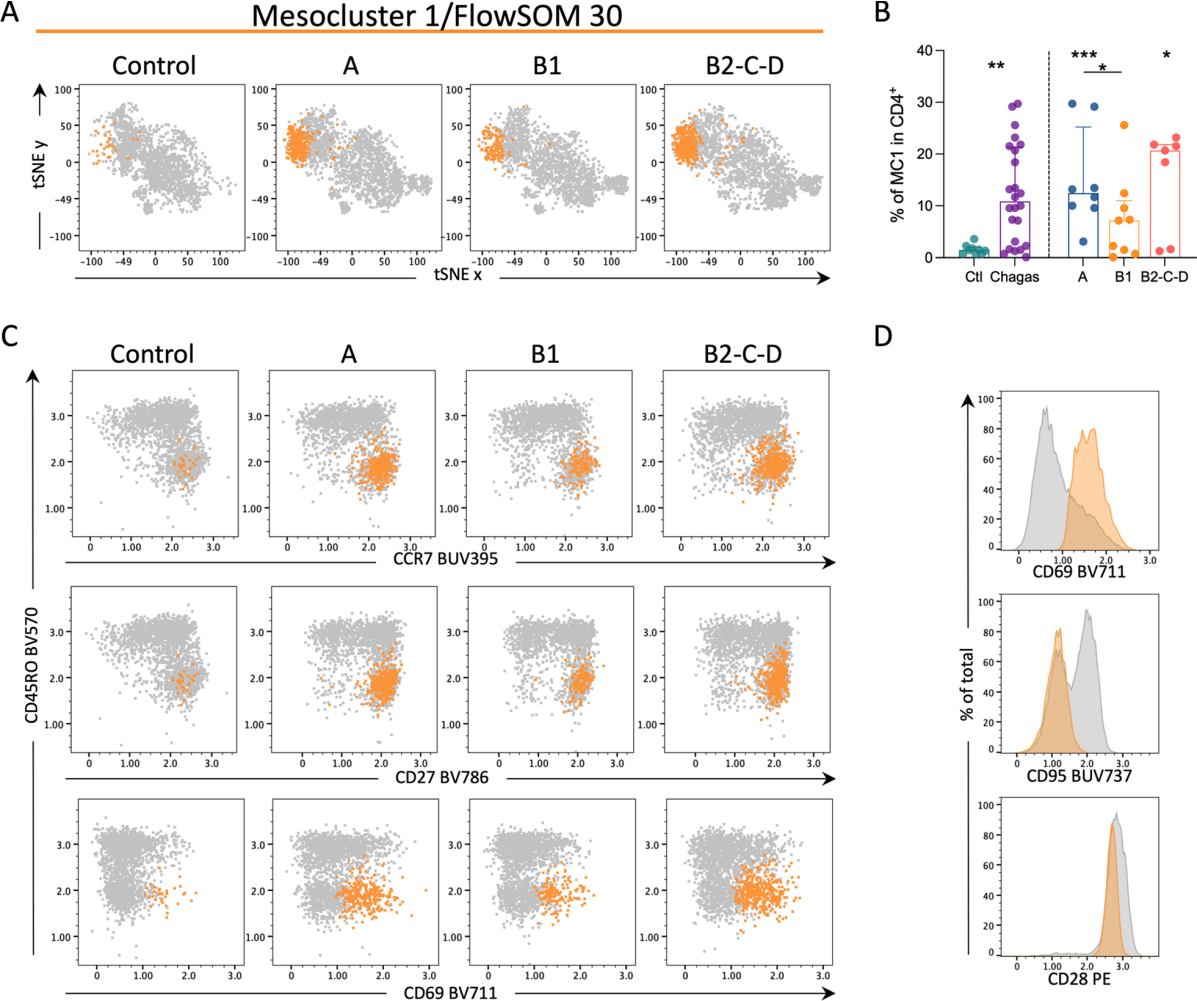
Activated subpopulations expressing CD69 are increased among naive CD4^+^ T cells in patients with Chagas disease. **A** tSNE plots depict CD4^+^ T cells in gray and the FlowSOM population number 30 (MC1) in yellow from controls and infected patients in different stages of Chagas disease (A, B1, B2-C-D). **B** Frequency of MC1 in CD4^+^ T cells. Bars represent median and interquartile range. Asterisks represent significant differences between the assigned group and controls. Asterisks over connecting lines represent significant differences between the assigned groups. *p < 0.05, **p < 0.01, ***p < 0.001. **C** Representative dot plots show the expression of CCR7, CD27, and CD69 (x-axis) by CD45RO (y-axis) from controls and infected patients in different stages of Chagas disease (A, B1, B2-C-D): CD4^+^ T cells in gray and MC1 in yellow. **D** Histograms represent the expression of CD69, CD95, and CD28 (x-axis) in CD4^+^ T cells in gray and the MC1 in yellow. In each histogram, the population represented in gray or yellow is scaled by the total events of each population (% of total, y-axis)

Metacluster 30 was topologically represented in one branch of the MST along with six clusters that were grouped as mesocluster 1 (MC1, Fig. 2A, B). The frequency of MC1 was increased in patients with Chagas disease compared to healthy controls (Fig. 3A and B). Higher frequencies were observed between controls and groups A and B2-C-D, while in group B1 five individuals displayed a higher proportion of these cells, but the group did not reach statistical significance (Fig. 3A, B). Although the frequency of T_N_ is comparable among the examined groups (Fig. 1B), their phenotype is different as defined by MC1 cluster, mainly composed of T_N_ with high expression of CD69 and CD28, but lacking CD95 expression (Fig. 3C, D).

**Fig. 4:**
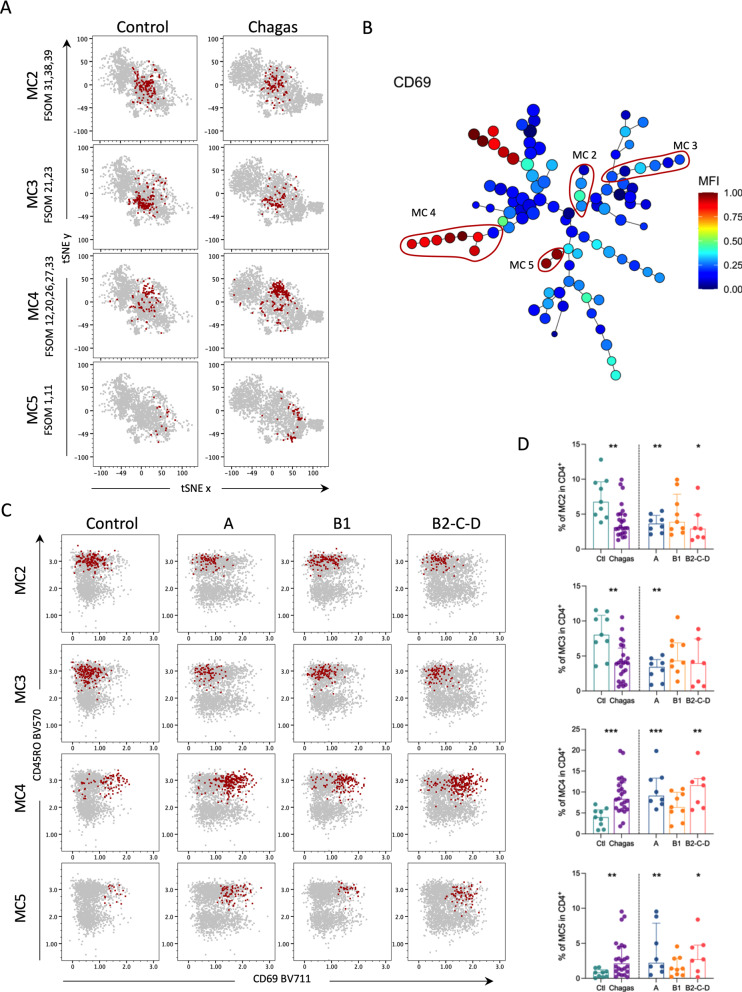
FlowSOM reveals unique memory CD4^+^ T cells defined by CD69 expression in patients with Chagas disease. **A** tSNE plots depict CD4^+^ T cells in gray and MC2 (FlowSOM 31, 38, 39), MC3 (FlowSOM 21, 23), MC4 (FlowSOM 12, 20, 26, 27, 33) and MC5 (FlowSOM 1, 11) in red from controls and infected patients with Chagas disease. **B** MST shows the differential expression of CD69, represented from blue to red indicating lower to higher expression, respectively. MC2-5 are highlighted by red circles. **C** Representative dot plots of the expression of CD69 (x-axis) by CD45RO (y-axis) from controls and infected patients in different stages of Chagas disease (A, B1, B2-C-D): CD4^+^ T cells are shown in gray and MC2-5, from the top to bottom, in red. **D** Frequencies of MC2-5 CD4^+^ T cells. Bars represent the median and interquartile range. Asterisks represent significant differences between the assigned group and controls. *p < 0.05, **p < 0.01, ***p < 0.001

Several metaclusters were differentially expressed between clinical forms and controls (Additional file 2: Fig. S2). These metaclusters showed similar expression patterns of specific markers (Fig. 2C), and clustered in the same branches in the MST. Thus, we grouped some of these metaclusters into mesoclusters 2, 3, 4, and 5 (Fig. 4A, B). All four mesoclusters were composed of T_CM_ and T_EM_ (Fig. 2A, C). MC2 and MC3 displayed low expression of CD69, while MC4 and MC5 displayed higher expression of this molecule (Fig. 4B, C). Frequencies of MC2 and MC3 were lower in patients compared to healthy controls, while MC4 and MC5 were expanded, mainly in clinical groups A and B2-C-D (Fig. 4D). To verify the validity of the unsupervised analysis, we performed a manual analysis of the expression of CD69 among the memory subpopulations of CD4^+^ T cells, which confirmed the higher expression of this marker in CCC patients (Additional file 3: Fig. S3). Interestingly, the frequency of CD69^+^CD4^+^ T cells was lower in group B1 compared to both group A and B2-C-D, although higher compared to controls. Regarding the memory compartments, the frequencies of CD69^+^CD4^+^ T cells in T_N_, T_CM_, and T_EM_ were lower in B1 compared to group A, and in T_N_ compared to group B2-C-D (Additional file 3: Fig. S3). Altogether, these findings revealed a CD69 signature in both naïve and memory T cells in Chagas patients.

Taken together, these data indicate a higher expression of CD69 in CD4^+^ T cells in patients with Chagas disease, especially in those in the indeterminate form (A) and established chronic cardiomyopathy (B2-C-D).

### Regulatory T cells expressing CD39 are decreased in mild CCC

**Fig. 5:**
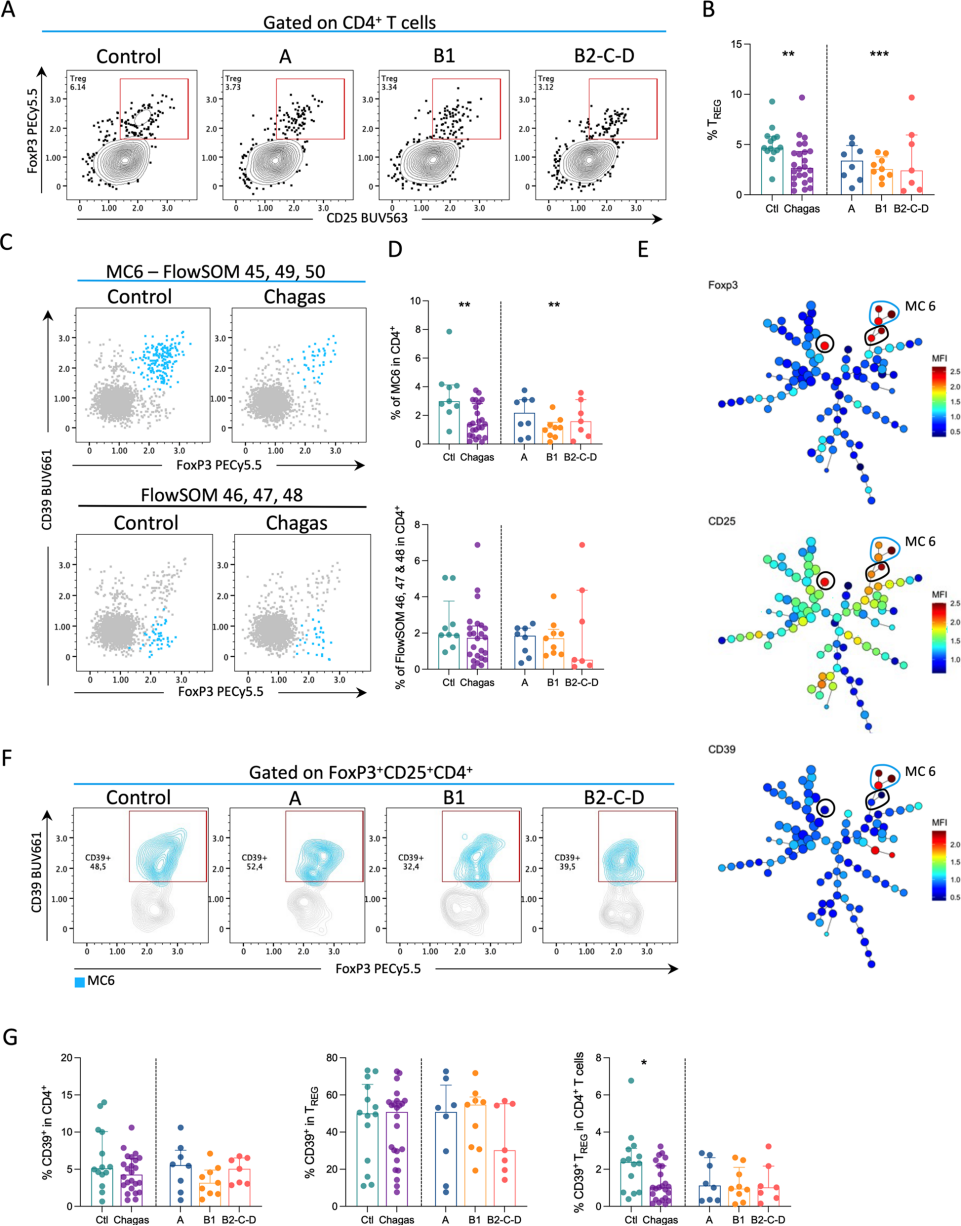
Regulatory T cells expressing CD39 are decreased in mild cardiomyopathy. Representative contour plots (**A**) and frequencies (**B**) of T_REG_, defined by the expression of FoxP3 and CD25, from controls and infected patients in different stages of Chagas disease (A, B1, B2-C-D). **C** Expression of CD39 and FoxP3 in cells from cluster MC6 (FlowSOM clusters 45, 49 and 50; top row) and combined FlowSOM clusters 46–48 (bottom row) between controls and patients are shown. Clusters are shown in blue and overlaid on total CD4^+^ T cells (grey). **D** The frequency of MC6 (top) and combined FlowSOM clusters 45, 49 and 50 (bottom) is shown for controls and patient groups. Bars represent the median and interquartile range. **E** MST shows the differential expression of FoxP3, CD25, and CD39, represented from blue to red indicating lower to higher expression, respectively. MC6 is highlighted by blue circles and FlowSOM populations 46, 47 and 48 by black circles. **F** Representative contour plots depict the expression of CD39 (blue) among T_REG_ (gray). **G** Frequency of CD39^+^ T cells in CD4^+^ T cells (left), frequency of CD39^+^ in T_REG_ (middle) and frequency CD39^+^T_REG_ in CD4^+^ T cells (right). Bars represent the median and interquartile range. Asterisks represent significant differences between Chagas’ patients and controls. *p < 0.05, **p < 0.01, ***p < 0.001

Regulatory T cells were distributed along with six clusters topologically located in two branches of the MST and with a very diverse expression pattern between metaclusters (Fig. 2). Among those metaclusters, differences between groups were observed in metaclusters 45, 49, and 50 (Additional file 2: Fig. S2), which are topologically located in the same branch, and therefore were grouped into MC6. Manual strategy showed a decreased frequency of T_REG_ in patients, especially within group B1 (Fig. 5A, B). Decreased T_REG_ frequency in patients with Chagas disease is mainly due to the decreased frequencies of T_REG_ expressing CD39, which was more pronounced in group B1 (Fig. 5C, D). Unsupervised clustering revealed a dichotomous expression of CD39 among MC6 and clusters 46, 47, and 48 (Fig. 5E, F). We confirmed the reduced frequencies of CD39^+^ T_REG_ among CD4^+^ T cells during Chagas disease compared to controls by manual gating analysis (Fig. 5G).

Together, these findings suggest a reduced regulatory potential in PBMC from patients with Chagas disease due to lower frequencies of T_REG_ expressing CD39.

### Granzyme B and perforin co-expressing cells are expanded during Chagas disease

**Fig. 6:**
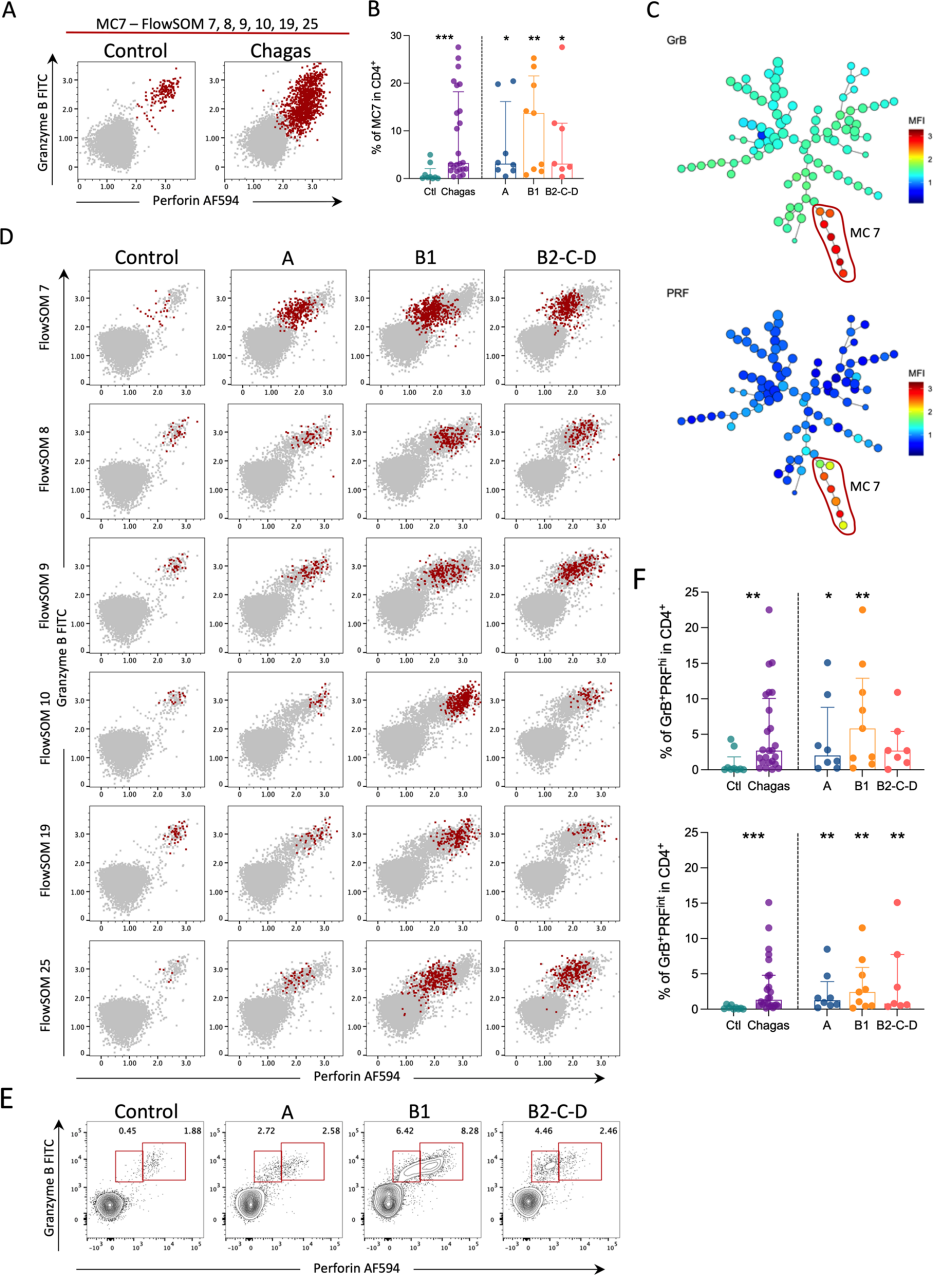
Granzyme B and perforin co-expressing cells are expanded during Chagas disease. Representative contour plots (**A**) and frequencies (**B**) depicting the expression of granzyme B and perforin in MC7 in controls (n = 9) and infected patients with Chagas disease (Chagas, n = 24). **C** MST shows the differential expression of granzyme B and perforin, represented from blue to red indicating lower to higher expression, respectively. MC7 is highlighted by a red circle. **D** Representative dot plots of the expression of perforin (x-axis) by granzyme B (y-axis) from controls and infected patients in different stages of Chagas disease (A, B1, B2-C-D). CD4^+^ T cells are colored in gray and each FlowSOM population part of MC7 individually in red. **E** Contour plots depict the intermediate and high expression of perforin in controls and infected patients in different stages of Chagas disease (A, B1, B2-C-D). **F** Bars represent the median and interquartile range. Asterisks represent significant differences between the assigned group and controls. *p < 0.05, **p < 0.01, ***p < 0.001

Metaclusters 7, 8, 9, 10, 19, and 25 were topologically close in MST and displayed a very similar expression pattern (Fig. 2A–C). In addition, all these metaclusters had increased frequencies in patients with Chagas disease (Additional file 2: Fig. S2). All these populations had a phenotype compatible with T_EM_, except for metacluster 9 that lacks the expression of CD45RO, and thus is mostly represented by T_EFF_ (Fig. 2A–C). Hence, we grouped these metaclusters into a MC7, which was increased in frequency among patients compared to controls (Fig. 6B) and is mainly defined by the expression of the cytotoxic granules granzyme B (GrB) and perforin (PFN) (Fig. 6A–C). A double pattern in the expression of an intermediary (GrB^+^PFN^int^) or high (GrB^+^PFN^hi^) levels of PFN was observed within cells expressing GrB (Fig. 6D and E). Both populations were increased in patients with Chagas disease and in the different clinical forms compared to controls, except for GrB^+^PFN^hi^ in group B2-C-D (Fig. 6F). CD4^+^ T cells expressing GrB and PFN are also known as cytotoxic CD4^+^ T cells. The increased frequencies of cytotoxic CD4^+^ T cells in patients with Chagas disease suggests a role of these cells in the pathogenesis of cardiomyopathy.

### Cytokine producing effector memory CD4^+^ T cells are responsive to antigenic recall increased during mild cardiac Chagas' disease

**Fig. 7:**
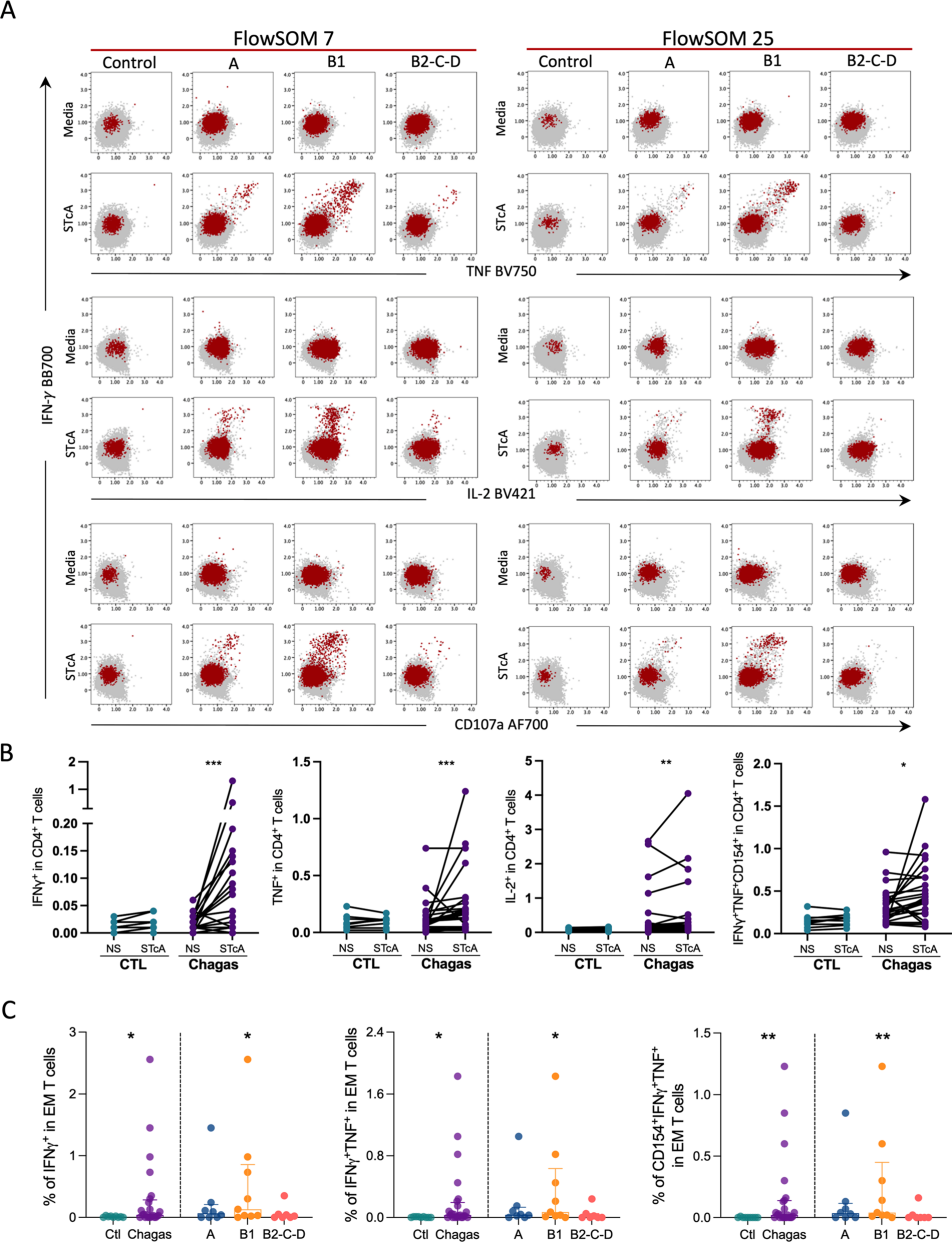
Cytokine producing effector memory CD4^+^ T cells are responsive to antigenic recall and are increased during mild cardiac Chagas disease. **A** Representative dot plots of the expression of TNF, IL-2, and CD107a (x-axis) by IFN-γ (y-axis) from controls and infected patients in different stages of Chagas disease (A, B1, B2-C-D), before and after the stimulation with soluble *T. cruzi* antigen (STcA). CD4^+^ T cells are colored in gray and FlowSOM populations 7 and 25 in red. **B** Frequencies of IFN-γ, TNF, IL-2, and CD154^+^TNF^+^IFN-γ^+^ cells in CD4^+^ T cells stimulated (STcA) or not (NS) with soluble *T. cruzi* antigen. Each dot represents an individual. Lines connect paired observations from the same individual. **C** Frequencies of IFN-γ, IFN-γ and TNF, IFN-γ and TNF and CD154 in effector memory (EM) CD4^+^ T cells. Bars represent the median and interquartile range. Asterisks represent significant differences between the assigned group and controls. *p < 0.05, **p < 0.01, ***p < 0.001

To evaluate the antigen-specific response of CD4^+^ T cells against *T. cruzi* soluble antigen (STcA), we assessed the expression of cytokines and inducible markers after in vitro stimulation. The induction of IFN-γ was observed in metaclusters 7 and 25, both within MC7, which is mainly composed of T_EM_ with an intermediary expression of PFN (Figs. 2A–C, 6D, 7A and Additional file 4: Fig. S4B). Expression of IFN-γ, IL-2, TNF and the coexpression of CD154, IFN-γ and TNF under antigen-specific stimulation was increased in patients compared to controls (Fig. 7B). Interestingly, higher activation upon STcA stimulation was observed in patients with mild cardiomyopathy (group B1) than controls (Fig. 7C). No relevant expression of IFN-γ or TNF was observed in metaclusters 8, 9, 10, and 19 (Additional file 4: Fig. S4). These results show the persistence of *T. cruzi*-specific T cells during mild cardiomyopathy.

## Discussion

We used high dimensional flow cytometry to assess the heterogeneity of CD4^+^ T cells from patients in various stages of Chagas disease. Clinical classification of patients is challenging due to the heterogeneous clinical presentation, and the difficulty to detect the very early myocardial injury. In this study, patients with Chagas disease were segregated in those who have no signs of cardiomyopathy (group A) from those with mild chronic cardiomyopathy (group B1) and patients with established chronic cardiomyopathy (group B2-C-D) [1, 22].

Unsupervised clustering revealed an increase of CD69^+^ cells among T_N_ (MC1) and memory (MC4 and MC5) CD4^+^ T cells in PBMC from patients with Chagas disease. CD69 is rapidly upregulated in CD4^+^ T cells after activation and is involved in their retention in lymph nodes due to CD69-mediated downregulation of sphingosine-1 phosphate receptor (S1PR1), which leads to very low proportions of CD69^+^ T cells in blood under homeostatic conditions [26, 27]. CD69 is constitutively co-expressed with CD103 on mucosal and skin resident memory T cells (T_RM_) [28]. Indeed, CD69 gene expression is upregulated in the heart from CCC patients compared to healthy controls [29, 30]. The absence of T_RM_ in the circulation suggests these cells are not prone to recirculate [28]. Thus, the expansion of CD69^+^CD4^+^ T cells during Chagas disease is probably not due to recirculation of T_RM_. Furthermore, previous data have described an antigen-experienced population, named stem cell memory T cells (T_SCM_), that displays strong multipotency and can give rise to all memory subsets [30, 31] and is characterized by expressing a naïve phenotype [32]. The hallmark of T_SCM_ is the expression of CD95, which is not observed in MC1 (Fig. 3D).

Although the expression of CD69 on naïve CD4^+^ T cells has been published in some pathological conditions, such as Graves’ disease and autoimmune thyroiditis [33], no reports were found in Chagas disease. Patients with the indeterminate clinical form displayed a higher frequency of CD69^+^CD4^+^ γδ T cells as compared to healthy individuals and CCC, either ex vivo or after stimulation with trypomastigote protein fraction [34]. In addition, CD69 expression was increased among CD25^high^CD4^+^ T cells from both indeterminate and CCC compared to healthy controls, either ex vivo or after stimulation with epimastigote antigen [35]. In our study, a consistent increase in the frequencies of CD69^+^CD4^+^ T cells among all T cell subsets was observed during Chagas disease. Notably, CD69 was the only marker analyzed able to distinguish B1 from A and B2-C-D.

The increased frequency of CD69^+^CD4^+^ T cells suggests a constant activation by remaining parasites or antigens that persist into the Chagas disease’s chronic phase [9]. Moreover, under certain conditions, CD69 expression may also be induced by IL-6, TNF, and IL-2 in naïve and memory T cells [36]. In this sense, either antigenic stimulation or chronic release of pro-inflammatory cytokines could induce and sustain an activated status among CD4^+^ T cells in patients with Chagas disease.

It has been suggested that pro-inflammatory mechanisms during cardiomyopathy are partially modulated by regulatory mechanisms that involve regulatory T cells [11, 19, 20, 37–39]. Here we found a decreased frequency of regulatory T cells, primarily due to lower numbers of CD39-expressing T_REG_, which was more pronounced among patients in the B1 group. Among regulatory T cells, those expressing CD39 are apparently more stable in inflammatory conditions and more efficient in immunosuppressive function [40, 41]. Furthermore, the suppressive function of CD39^+^ T_REG_ has been studied in different diseases. Decreased frequencies of CD39^+^ T_REG_ are associated with inflammatory diseases such as multiple sclerosis, while increased frequencies are found in human colorectal cancer allowing cell proliferation [42, 43]. Regulatory T cells from CCC patients were previously reported as not being able to suppress PBMC proliferation [44]. Our findings also suggest that chronic Chagas disease progresses with loss of regulatory function and an increased pro-inflammatory signature in established cardiac disease. The contrasting results found by others [35, 37] might be explained by the differences in the immunophenotyping strategy and the ex vivo approach versus the response induced by antigenic stimulation. In the present work, T_REG_ were analyzed ex vivo and were defined by the expression of FoxP3 and CD25.

Here, GrB^+^PFN^+^CD4^+^ T cells were expanded in patients with Chagas disease compared to controls. Previous reports have shown an expansion of cytotoxic CD4^+^ T cells during Chagas disease [45–47]. CD4^+^ T cells with high expression of cytotoxic granules may be linked to apoptosis induction in, but not limited to, antigen-presenting cells [48]. Importantly, MHC-II is upregulated in heart endothelial cells from CCC patients, suggesting a potential role of cytotoxic CD4^+^ T cells in directly induced death of these cells [49]. Several different phenotypes of cytotoxic CD4^+^ T cells have been described and are considered to add protection during viral infections and cancer [48, 50]. CD45RA^+^CCR7^−^CX3CR1^+^CD4^+^ T cells expressing cytotoxic granules accumulate with multiple infections by Dengue virus [50]. Compatible with this phenotype, we described the metacluster 9 that was expanded in patients with Chagas disease, express a T_EFF_ phenotype (CD45RO^−^CCR7^−^) and co-express the cytotoxic granules GrB and PFN, thus suggesting that these cells exhibit the same function.

CD4^+^ T cells also produce several cytokines during chronic Chagas disease [51, 52]. Increased responses were observed in patients with Chagas disease, with the B1 group displaying the higher proportion of responders and the higher frequencies of IFN-γ^+^ activated CD4^+^ T cells, mainly among T_EM_. Multifunctional CD4^+^ T cells were observed to a lesser extent, and most of the responding cells co-express IFN-γ and TNF while lacking the expression of IL-2. Others have found a predominance of CD4^+^ T cells producing a single cytokine (IFN-γ, IL-2, or TNF) when using *T. cruzi* amastigote and trypomastigotes antigens [46, 53]. A higher frequency of IFN-γ^+^IL-2^−^CD4^+^ T cells, a phenotype associated with low parasite load and proliferation, has also been shown in patients in the indeterminate form compared to CCC [53].

Noteworthy, the antigen-specific CD4^+^ T cells producing cytokines were mostly represented by metaclusters 7 and 25, co-expressing GrB and PFN. Indeed, a GrB^+^PFN^+^IFN-γ^+^CD4^+^ T cell population was expanded in Chagas’ patients [46], suggesting that this population displays a role in CCC. Notably, host cytotoxic granules were shown to induce the direct killing of *T. cruzi* [54]. Altogether, these findings suggest a potential role of cytotoxic CD4^+^ T cells in chronic cardiomyopathy in Chagas disease, which may be inducing cell death of *T. cruzi* infected cardiomyocytes and endothelial cells, and producing cytokines that help effector functions. Whether their role is protective or detrimental in promoting cardiomyopathy is still unknown.

Finally, B1 patients displayed a unique signature of CD4^+^ T cell populations during Chagas disease. The expansion of cytokine-producing CD4^+^ T cells with cytotoxic activity, contemporaneously with contraction of CD39^+^ T_REG_, represented by mesocluster 6, suggests an imbalance of pro-inflammatory/regulatory responses. This imbalance may be associated with the cardiomyopathy progression initially observed in B1 patients. Furthermore, B1 has a unique expression of CD69 compared to other forms of Chagas disease, more pronounced among naïve T cells. Thus, the T cell signature observed in mild cardiomyopathy is expected due to more active myocarditis and tissue remodeling than patients with the indeterminate form. Indeed, IFN-γ-related chemokines are increased in B1 than indeterminate patients [14]. Moreover, the IFN-γ effector signature observed in B1 patients may favor the control of parasite load. Considering that persistent parasitemia may contribute to cardiomyopathy progression, we hypothesize that persistent antigenic stimulation slowly leads to the accumulation of certain effector T cells that promote lesion in the heart as observed in B2-C-D.

This is the first study using high-dimensional flow cytometry in the context of Chagas disease. The cell signatures described here revealed an imbalance of the inflammatory/regulatory response which is more pronounced during mild cardiomyopathy. Our results provide new insights on the pathogenesis and development of different clinical forms of Chagas disease. Additionally, we propose the combination of the markers here described as a supplementary tool for the clinical management of patients.

## Supplementary Information

**Additional file 1: Fig. S1.** tSNE plots depict CD4^+^ T cell subpopulations (TREG, FoxP3^+^CD25^+^; central memory, CM, CCR7^+^CD45RO^+^; effector memory, EM, CCR7^−^CD45RO^+^; effector, Eff, CCR7^−^CD45RO^−^; naïve, Nv, CCR7^+^CD45RO^−^) (**A**) and the 50 FlowSOM populations (**B** and** C**) from uninfected controls (Ctl, n = 9) and infected patients in different stages of Chagas disease: A (indeterminate, n = 8), B1 (mild cardiomyopathy, n = 9), B2-C-D (established cardiomyopathy, n = 7). Plots are normalized to represent the same number of events in each tSNE. Each metacluster is represented by a single color over density lines representing the bulk of CD4^+^ T cells (**C**).

**Additional file 2: Fig. S2.** Frequencies of each FlowSOM population in CD4^+^ T cells. Bars represent the median and interquartile range. Asterisks represent significant differences between the assigned group and controls. Asterisks over connecting lines represent significant differences between the assigned groups. *p < 0.05, **p < 0.01, ***p < 0.001

**Additional file 3: Fig. S3.** Frequencies of CD69 in CD4^+^ T cells and their memory compartments. CD4^+^ T cell subpopulations defined by the expression of CD45RO and CCR7 or CD27: naïve, CCR7^+^CD45RO^−^ or CD27^+^CD45RO^−^); central memory, CCR7^+^CD45RO^+^ or CD27^+^CD45RO^+^; effector memory, CCR7^−^CD45RO^+^ or CD27^−^CD45RO^+^; effector, CCR7^−^CD45RO^−^ or CD27^−^CD45RO^−^. Bars represent the median and interquartile range. Asterisks represent significant differences between the assigned group and controls. Asterisks over connecting lines represent significant differences between the assigned groups. *p < 0.05, **p < 0.01, ***p < 0.001, ****p < 0.0001

**Additional file 4: Fig. S4.** Frequencies of IFN-γ^+^ cells in FlowSOM populations 7, 8, 9, 10, 19 and 25 stimulated (STcA) or not (NS) with soluble *T. cruzi* antigen. Each dot represents an individual. Lines connect paired observations from the same individual. Asterisks represent significant differences between unstimulated and stimulated PBMC in each group. *p < 0.05, **p < 0.01, ***p < 0.001.

**Additional file 5: Table S1.** Clinical and echocardiographic characteristics of the study population. Values are expressed as mean and standard deviation (mean ± SD) or absolute number. LVEF, left ventricular ejection fraction; LVEDD, left ventricular end-diastolic diameter. *P = value for comparison between the groups (Kruskal-Wallis test). *Missing data for 1 participant.

**Additional file 6: Table S2.** List of antibodies and reagents used.

## Data Availability

The datasets during and/or analyzed during the current study available from the corresponding author on reasonable request.
