# Supplementary figures and images for: Chagasic cardiomyopathy is marked by a unique signature of activated CD4+ T cells
Source: J Transl Med. 2022 Nov 30;20:551. doi: 10.1186/s12967-022-03761-5 (PMC9708147; doi:10.1186/s12967-022-03761-5)

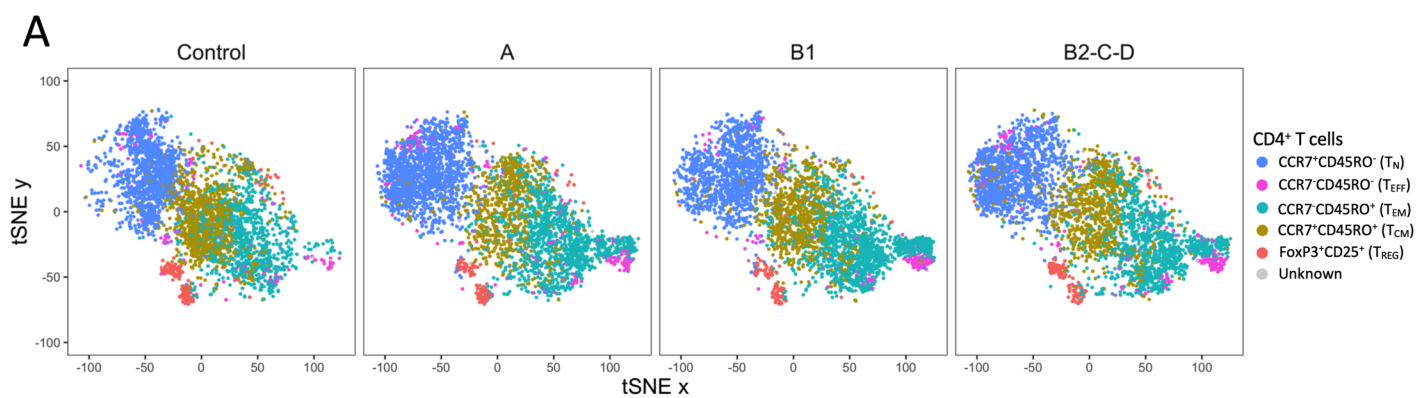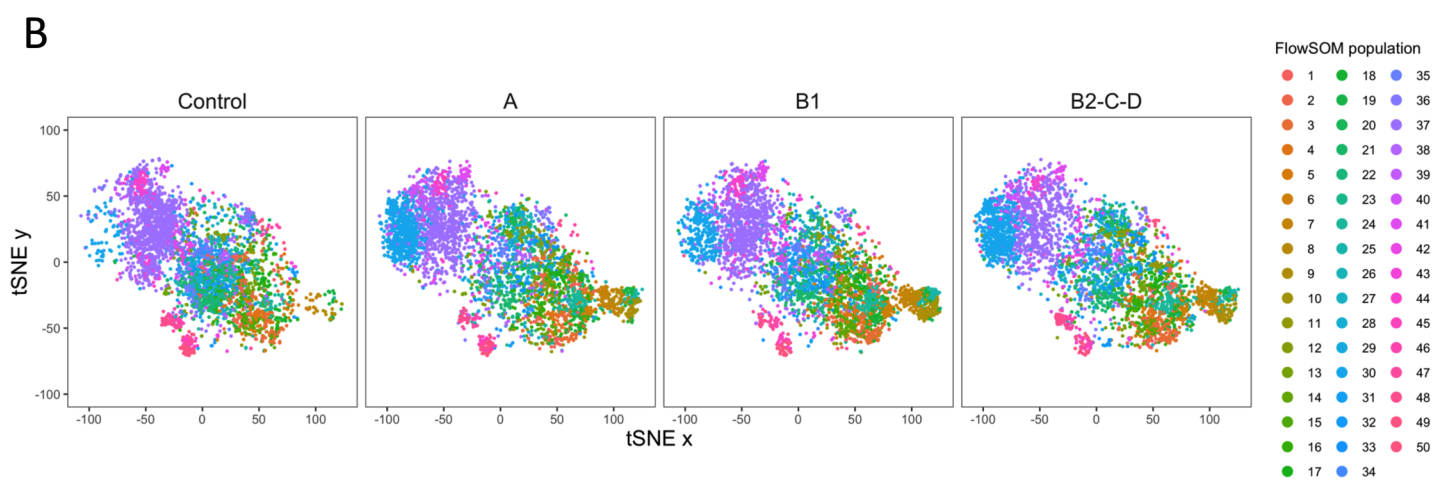

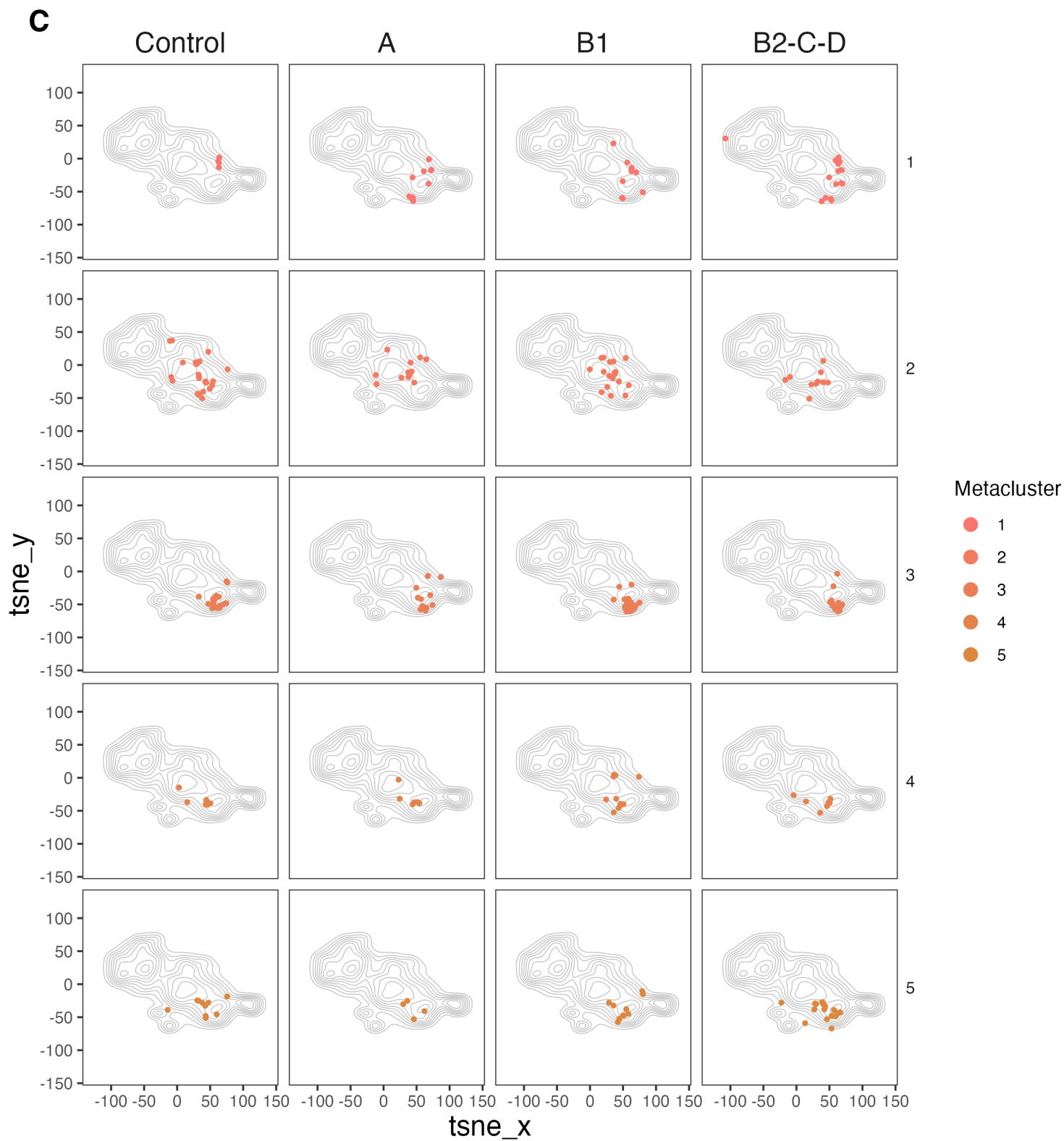

**C**

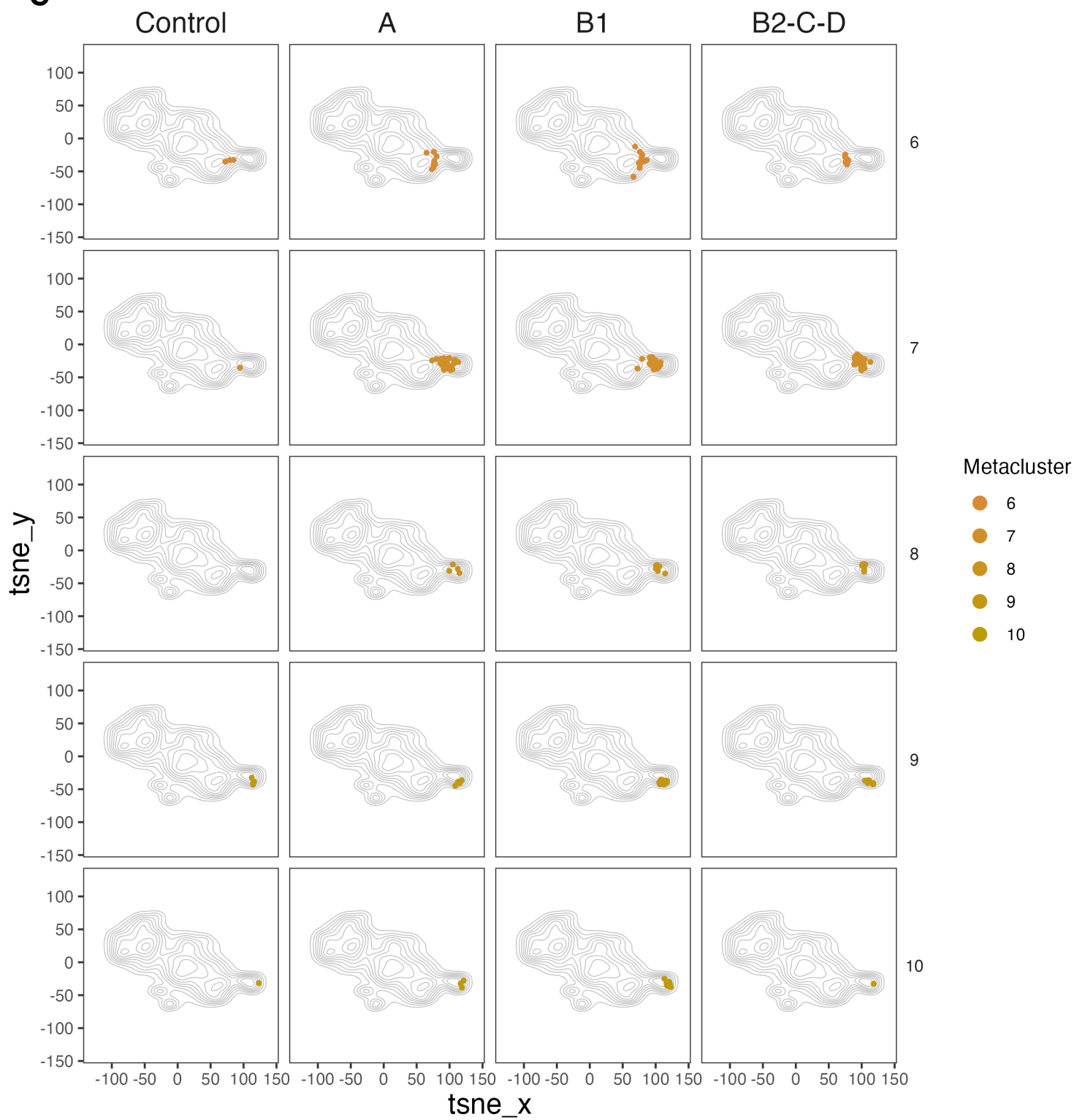

**C**

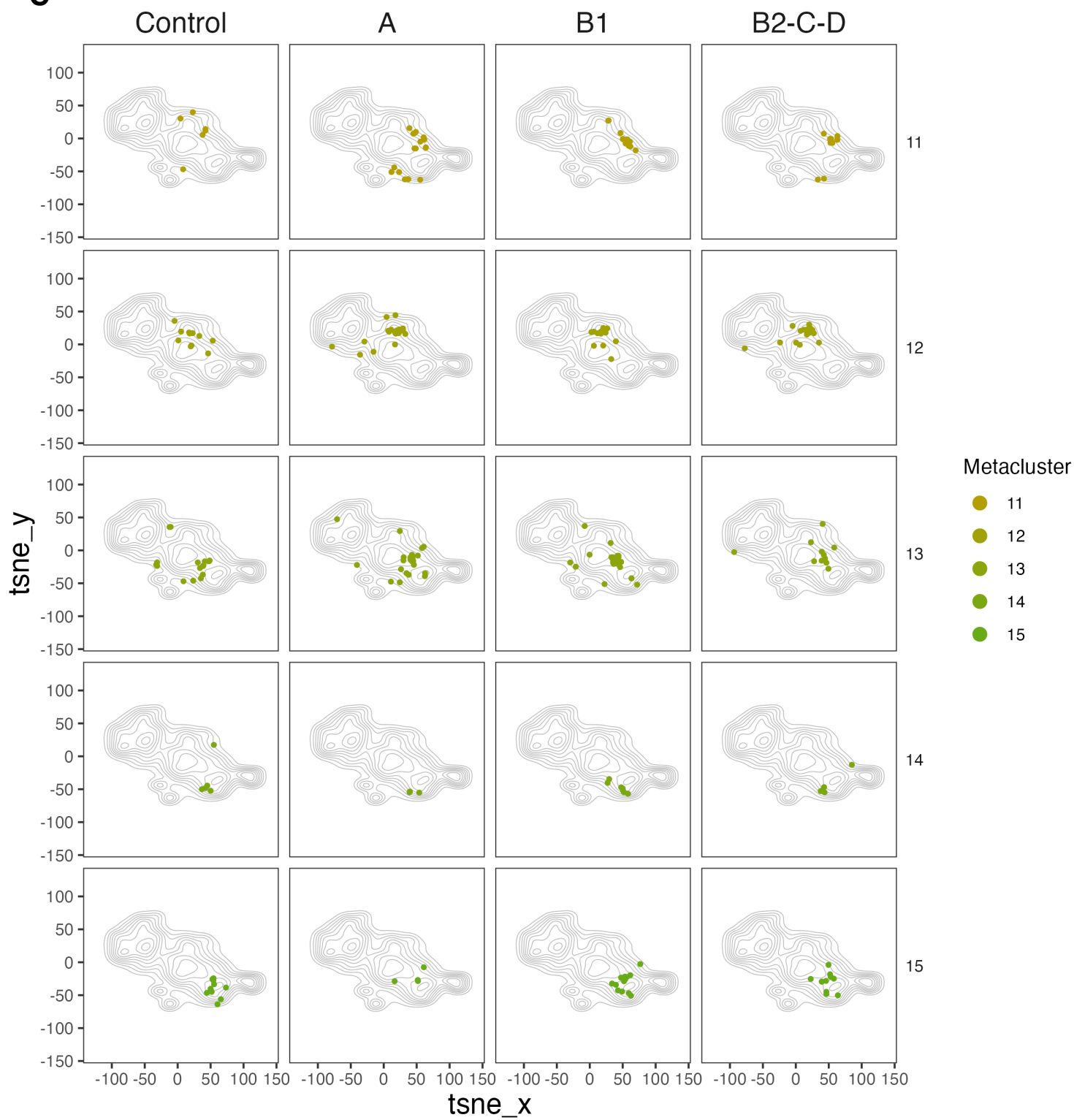

**C**

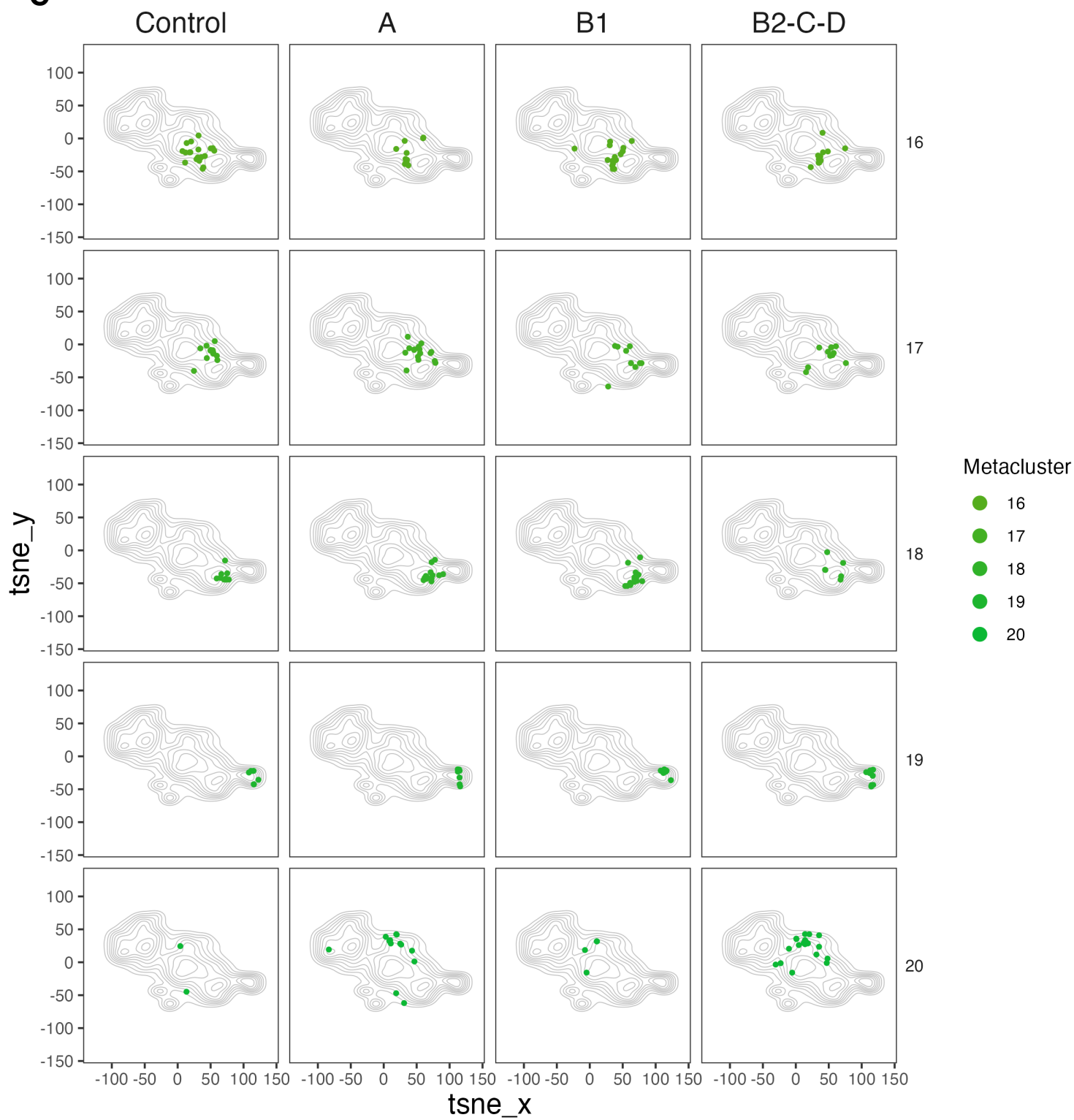

**C**

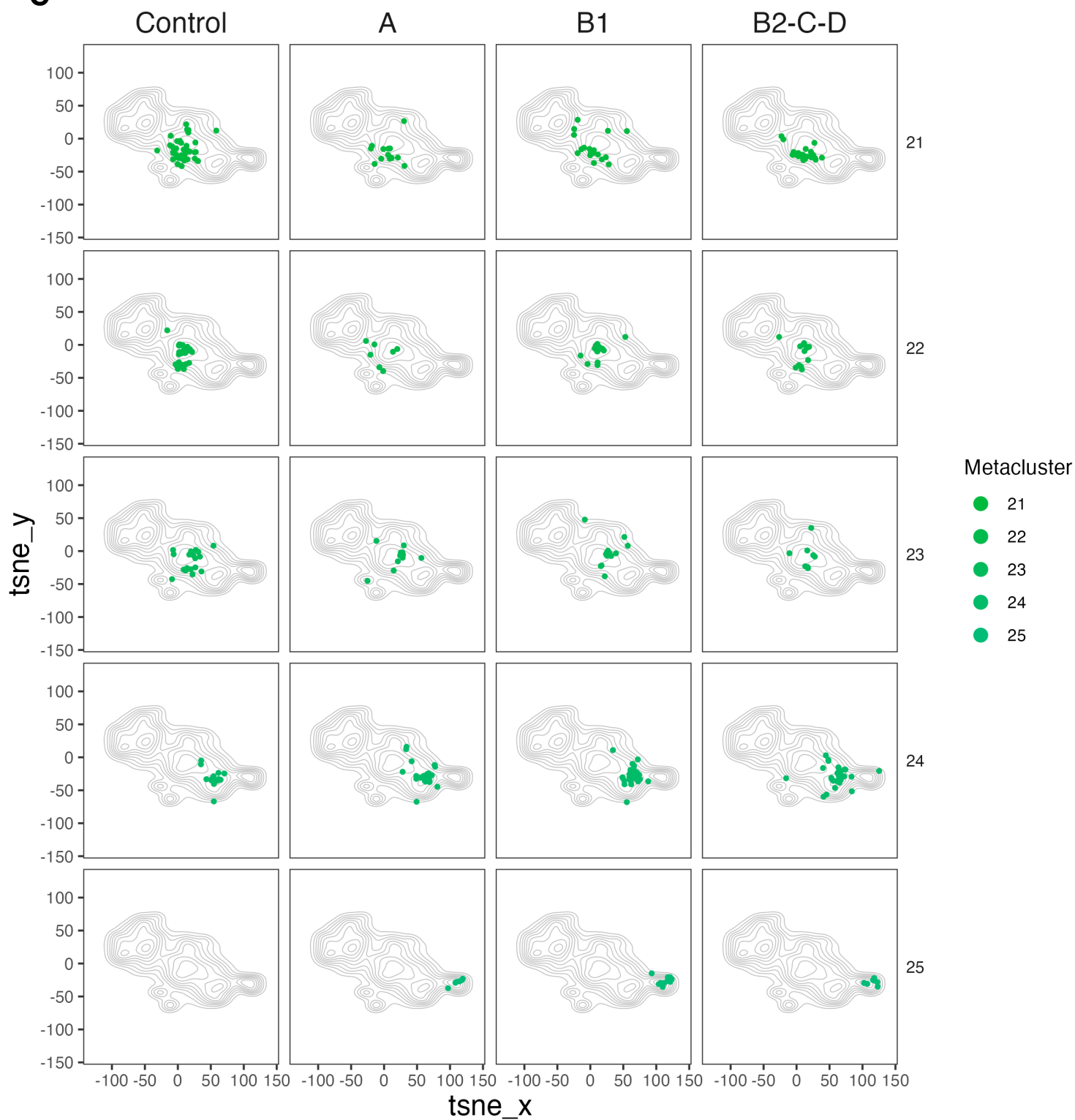

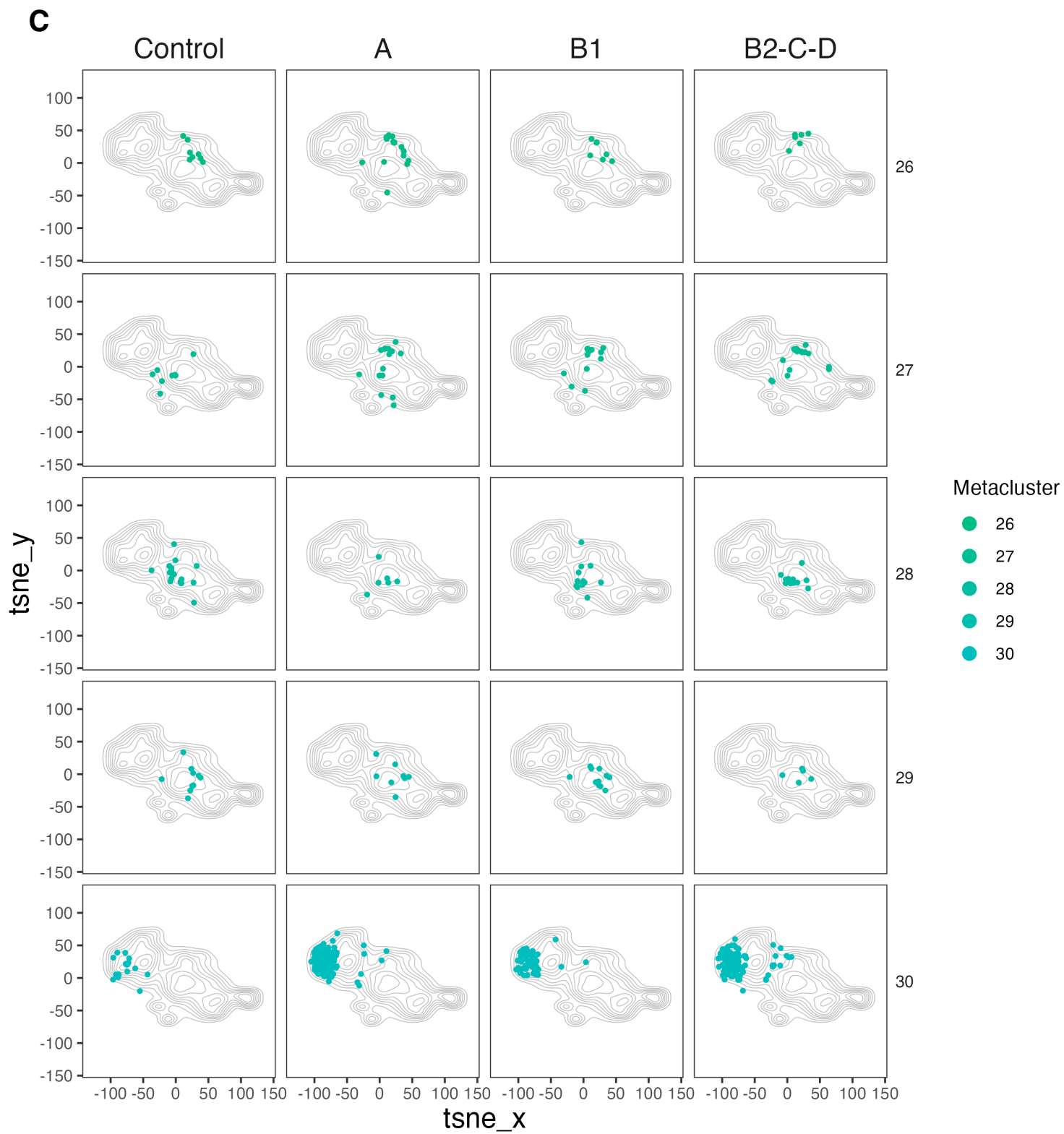

**C**

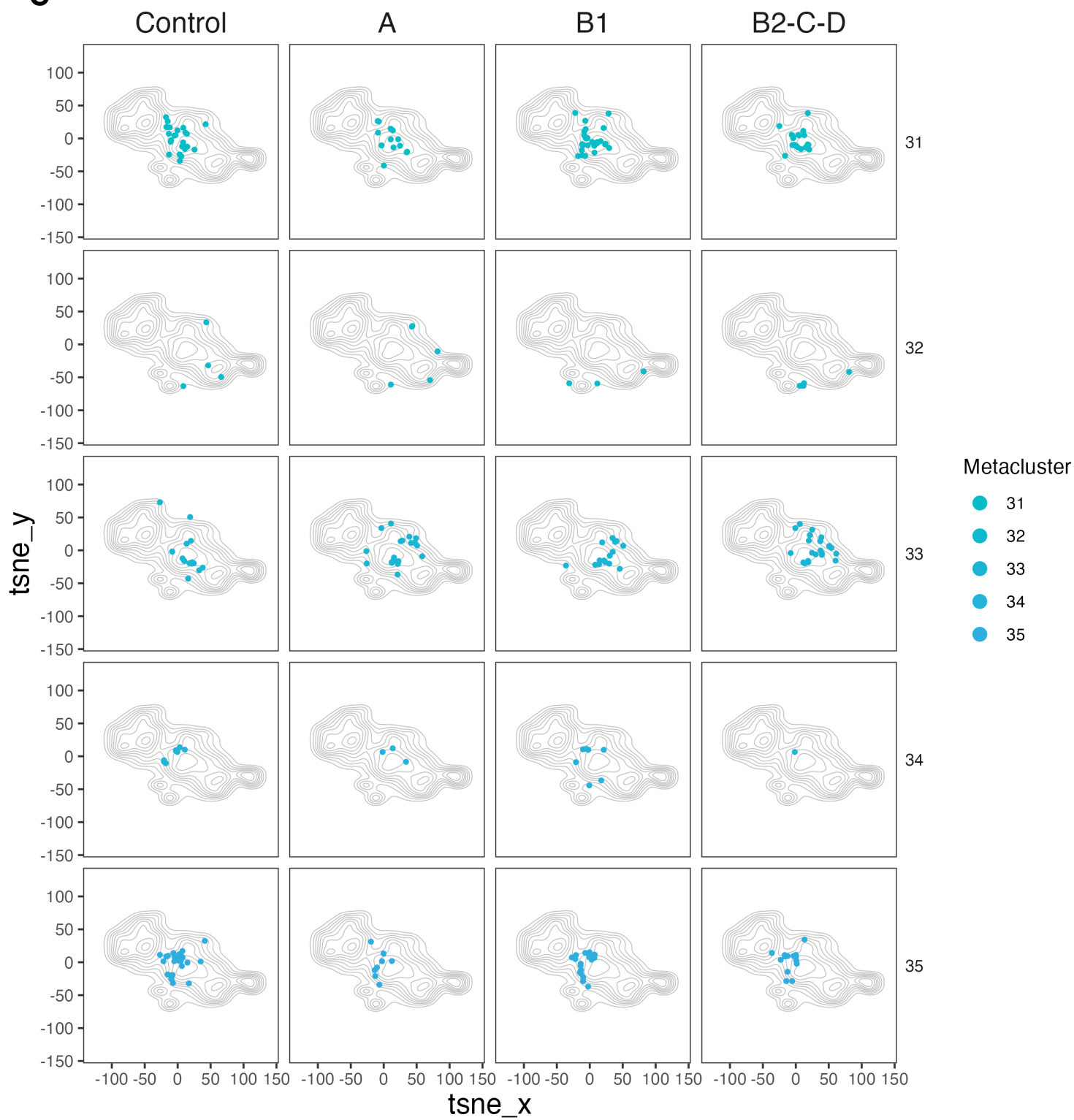

**C**

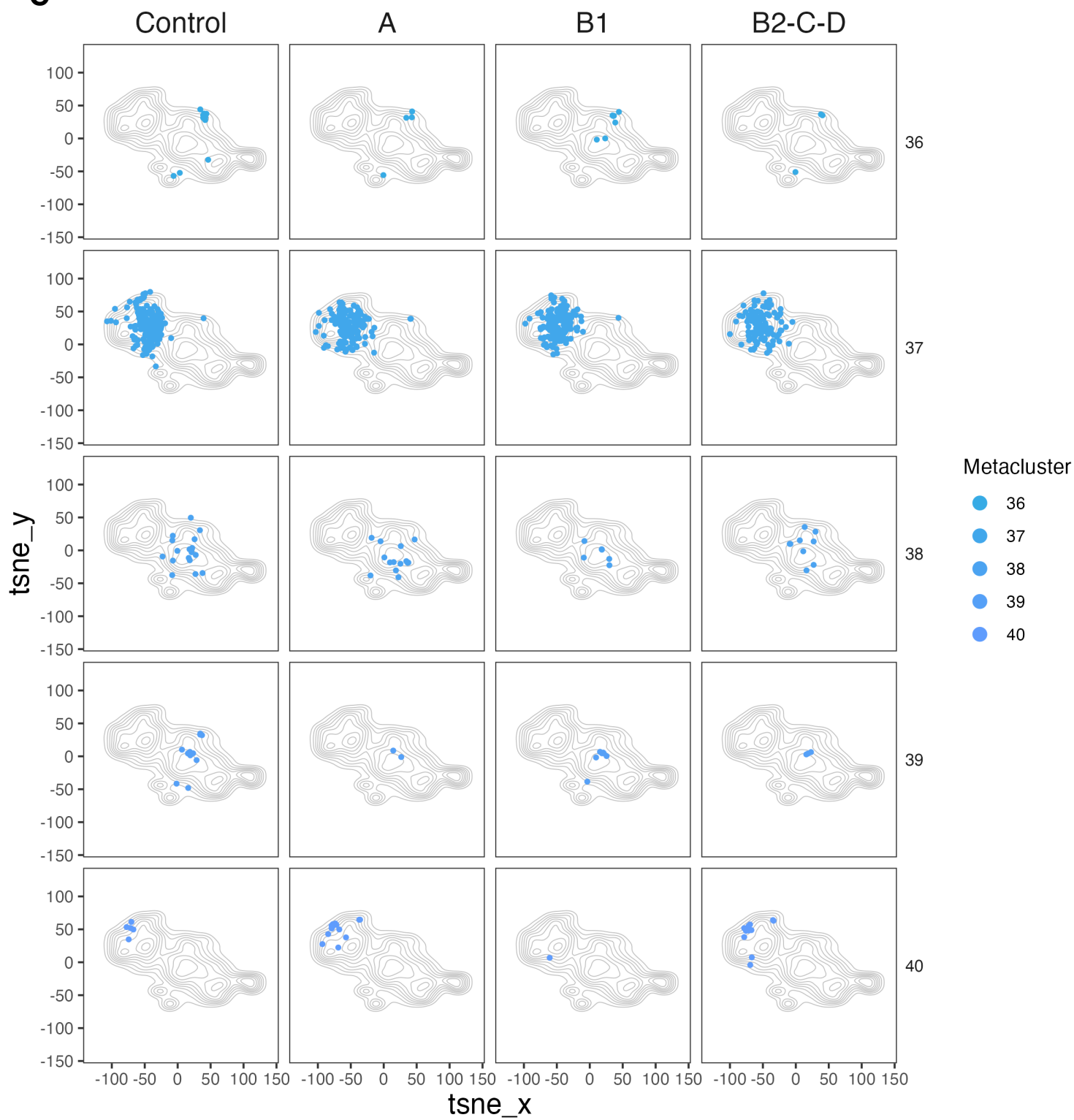

C

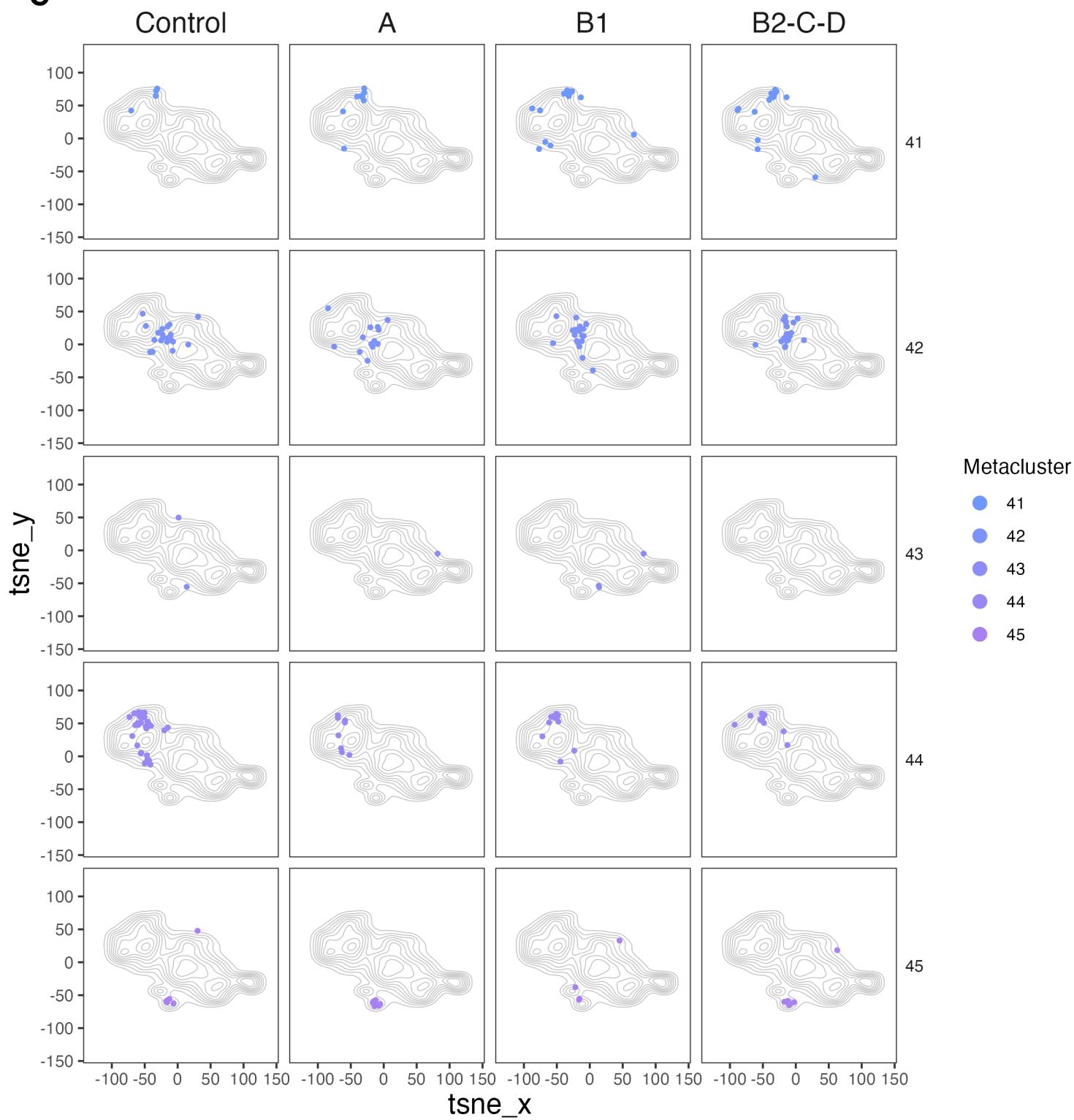

C

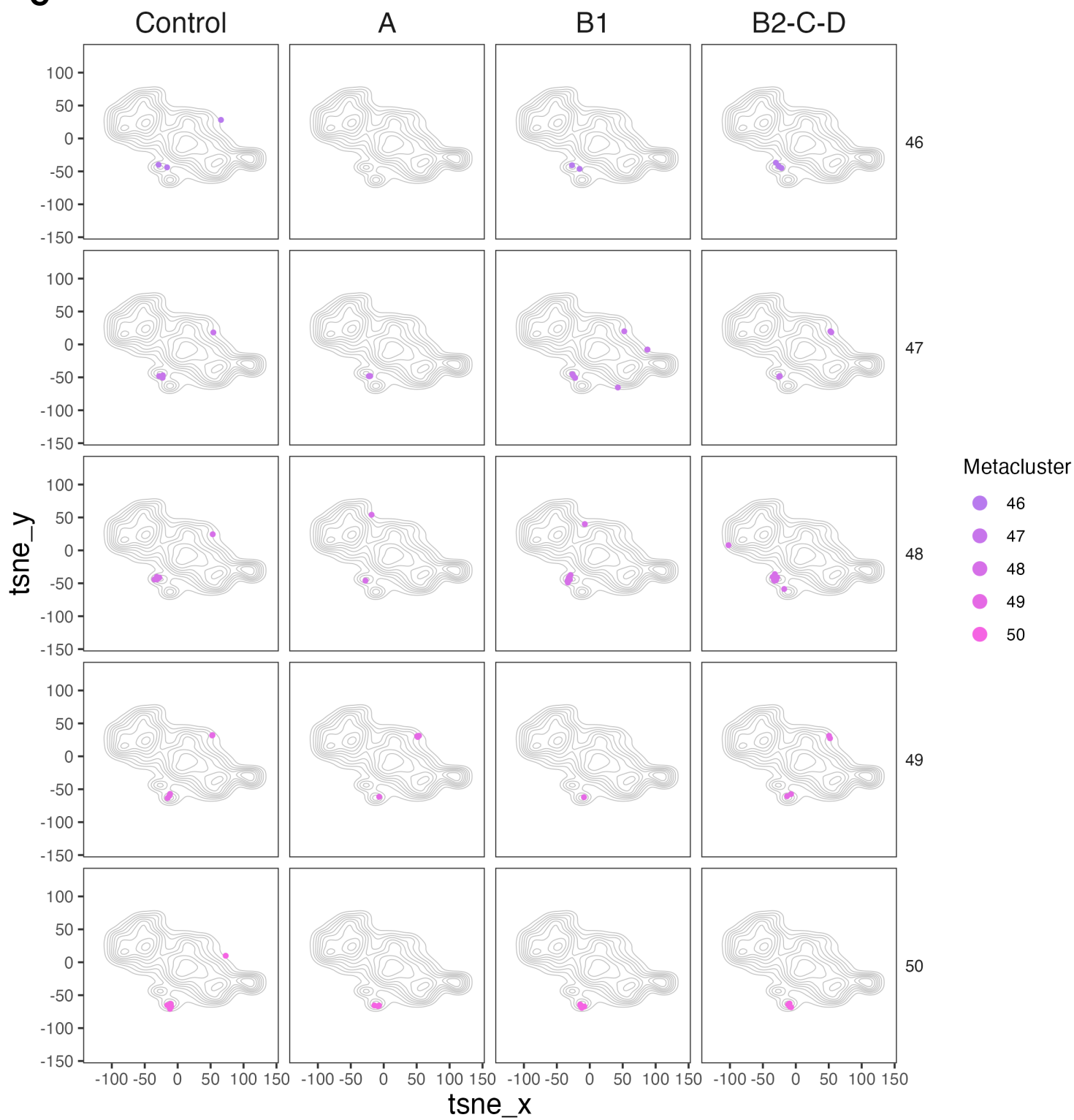

Supplement: Supplementary file 1 — Additional file 1: Fig. S1. tSNE plots depict CD4+ T cell subpopulations (TREG, FoxP3+CD25+; central memory, CM, CCR7+CD45RO+; effector memory, EM, CCR7−CD45RO+; effector, Eff, CCR7−CD45RO−; naïve, Nv, CCR7+CD45RO−) (A) and the 50 FlowSOM populations (B and C) from uninfected controls (Ctl, n = 9) and infected patients in different stages of Chagas disease: A (indeterminate, n = 8), B1 (mild cardiomyopathy, n = 9), B2-C-D (established cardiomyopathy, n = 7). Plots are normalized to represent the same number of events in each tSNE. Each metacluster is represented by a single color over density lines representing the bulk of CD4+ T cells (C). [file 12967_2022_3761_MOESM1_ESM.pdf]

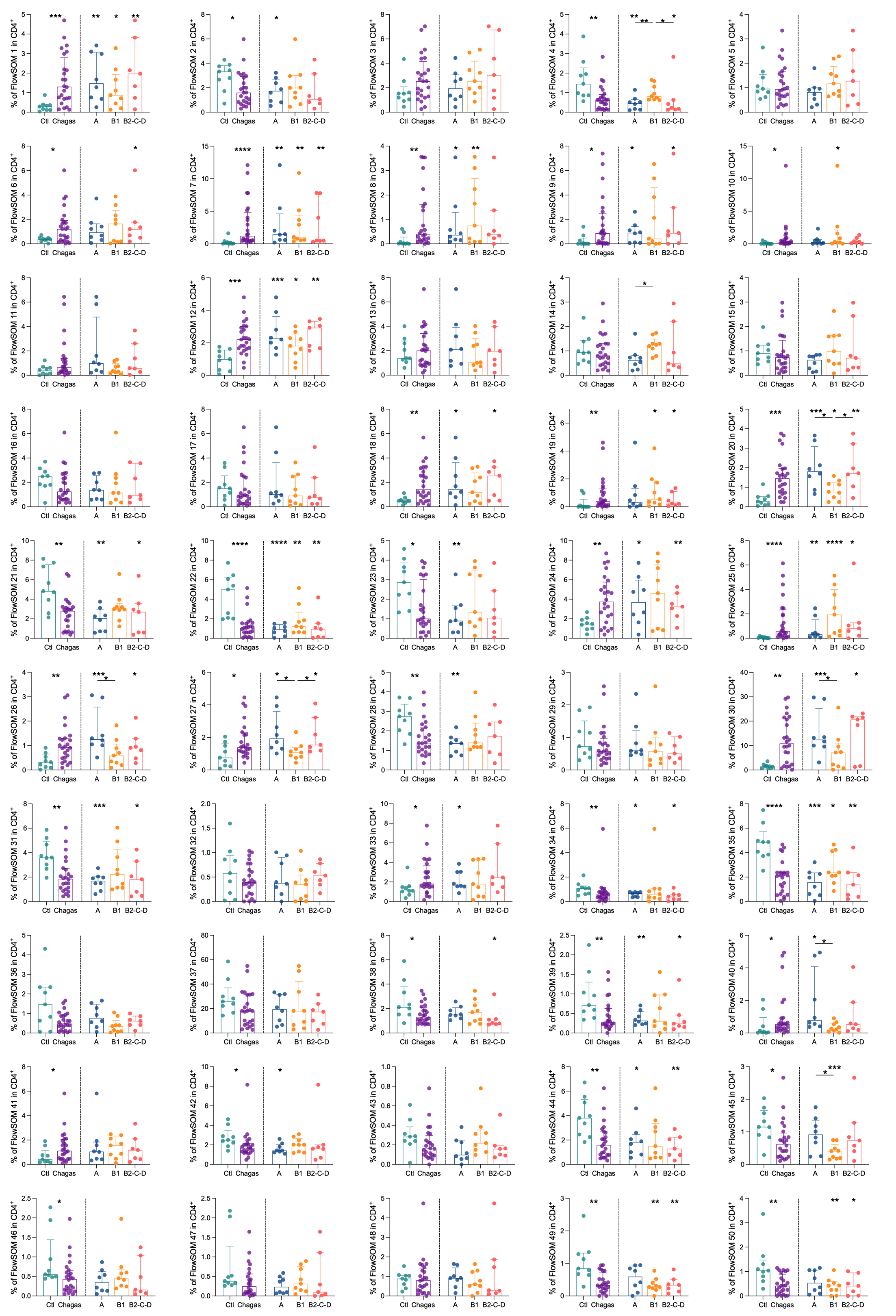

Supplement: Supplementary file 2 — Additional file 2: Fig. S2. Frequencies of each FlowSOM population in CD4+ T cells. Bars represent the median and interquartile range. Asterisks represent significant differences between the assigned group and controls. Asterisks over connecting lines represent significant differences between the assigned groups. *p < 0.05, **p < 0.01, ***p < 0.001 [file 12967_2022_3761_MOESM2_ESM.tiff]

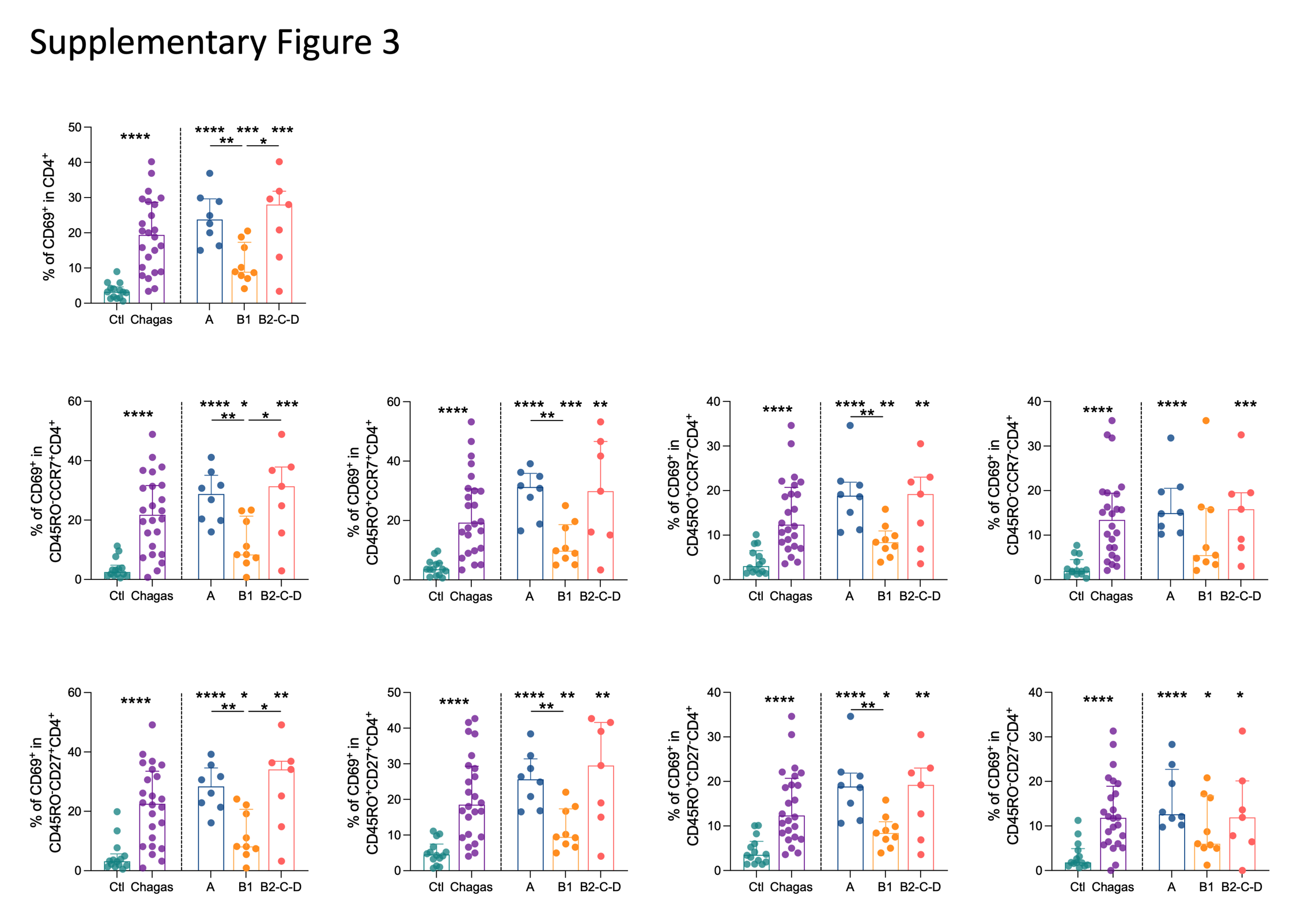

Supplement: Supplementary file 3 — Additional file 3: Fig. S3. Frequencies of CD69 in CD4+ T cells and their memory compartments. CD4+ T cell subpopulations defined by the expression of CD45RO and CCR7 or CD27: naïve, CCR7+CD45RO− or CD27+CD45RO−); central memory, CCR7+CD45RO+ or CD27+CD45RO+; effector memory, CCR7−CD45RO+ or CD27−CD45RO+; effector, CCR7−CD45RO− or CD27−CD45RO−. Bars represent the median and interquartile range. Asterisks represent significant differences between the assigned group and controls. Asterisks over connecting lines represent significant differences between the assigned groups. *p < 0.05, **p < 0.01, ***p < 0.001, ****p < 0.0001 [file 12967_2022_3761_MOESM3_ESM.tiff]

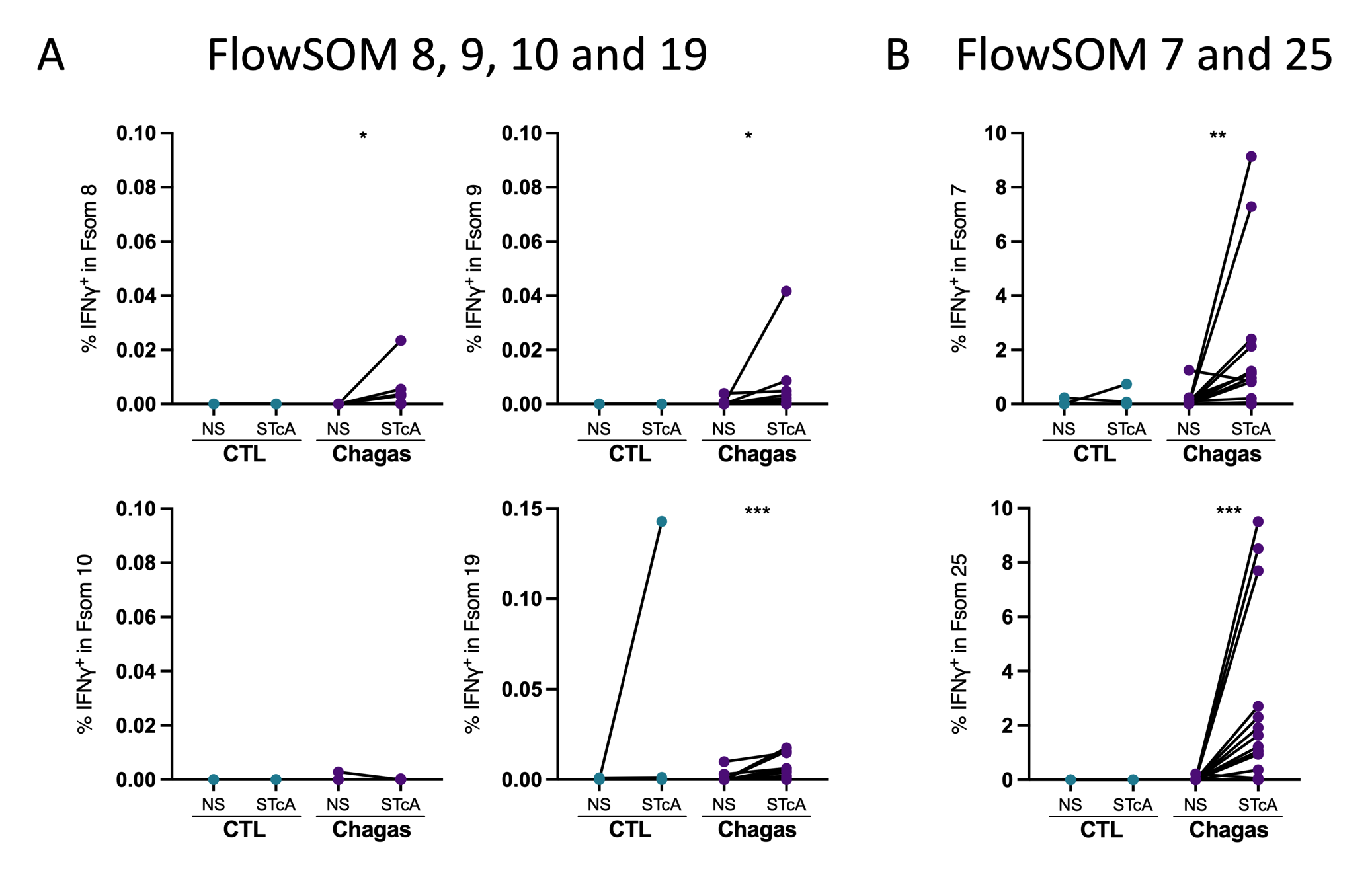

Supplement: Supplementary file 4 — Additional file 4: Fig. S4. Frequencies of IFN-γ+ cells in FlowSOM populations 7, 8, 9, 10, 19 and 25 stimulated (STcA) or not (NS) with soluble T. cruzi antigen. Each dot represents an individual. Lines connect paired observations from the same individual. Asterisks represent significant differences between unstimulated and stimulated PBMC in each group. *p < 0.05, **p < 0.01, ***p < 0.001. [file 12967_2022_3761_MOESM4_ESM.tiff]
