# Supplementary material for: Chagasic cardiomyopathy is marked by a unique signature of activated CD4+ T cells
Source: J Transl Med. 2022 Nov 30;20:551. doi: 10.1186/s12967-022-03761-5 (PMC9708147; doi:10.1186/s12967-022-03761-5)
Supplement: Supplementary file 5 — Additional file 5: Table S1. Clinical and echocardiographic characteristics of the study population. Values are expressed as mean and standard deviation (mean ± SD) or absolute number. LVEF, left ventricular ejection fraction; LVEDD, left ventricular end-diastolic diameter. *P = value for comparison between the groups (Kruskal-Wallis test). *Missing data for 1 participant. [file 12967_2022_3761_MOESM5_ESM.pdf]

	Indeterminate	Chagas cardiomyopathy		Controls	P-value*
	A (n=8)	B1 (n=9)	B2/C/D (n=7)	(n = 14)	
Age, yr	54 ± 10.2	50 ± 15.2	52 ± 9.5	41.6 ± 16.4	0.3574
Female/Male	4/4	5/4	2/5	5/8*	
LVEF (%)	68 ± 3.2	55.2 ± 15.0	38 ± 9.9	-	0.0001
LVEDD (mm)	45.6 ± 3.4	56.5 ± 8.1	67 ± 7.3	-	<0.0001

Table S1. Clinical and echocardiographic characteristics of the study population. Values are expressed as mean and standard deviation (mean ± SD) or absolute number. LVEF, left ventricular ejection fraction; LVEDD, left ventricular end-diastolic diameter. *P=value for comparison between the groups (Kruskal-Wallis test). *Missing data for 1 participant.
