# Supplementary material for: Chagasic cardiomyopathy is marked by a unique signature of activated CD4+ T cells
Source: J Transl Med. 2022 Nov 30;20:551. doi: 10.1186/s12967-022-03761-5 (PMC9708147; doi:10.1186/s12967-022-03761-5)
Supplement: Supplementary file 6 — Additional file 6: Table S2. List of antibodies and reagents used. [file 12967_2022_3761_MOESM6_ESM.docx]

Supplementary table 2: List of antibodies and reagents used.

| Specificity | Fluorochrome | Clone | Catalog | Manufacturer | Dilution | Staining |
| --- | --- | --- | --- | --- | --- | --- |
| Granzyme B | FITC | GB11 | 515403 | Biolegend | 1:160 | Intracellular |
| Tbet | BB630 | 4B10 | custom | BD Biosciences | 1:160 | Intracellular |
| IL-13 | BB660 | JES10-5A2 | 624295 | BD Biosciences | 1:320 | Intracellular |
| IFN-γ | BB700 | B27 | 566394 | BD Biosciences | 1:1280 | Intracellular |
| RORγt | BB790 | Q21-559 | custom | BD Biosciences | 1:160 | Intracellular |
| CD28 | PE | CD28.2 | 555729 | BD Biosciences | 1:10 | Surface |
| Perforin | Ax594 | B-D48 | 854.950.000BU | Diaclone | 1:320 | Intracellular |
| TCR-γδ | PE-Cy5 | B1 | 624350 | BD Biosciences | 1:20 | Surface |
| FoxP3 | PE-Cy5.5 | PCH101 | 35-4776-42 | Thermo Fisher Scientific | 1:40 | Intracellular |
| IL-22 | PE-Cy7 | 22URTI | 25-7229-42 | Thermo Fisher Scientific | 1:80 | Intracellular |
| IL-21 | Ax647 | 3A3-N2.1 | 560493 | BD Biosciences | 1:20 | Intracellular |
| CD107a | Ax700 | H4A3 | 561340 | BD Biosciences | 1:80 | During stimulation |
| CD3 | APC-H7 | SK7 | 624347 | BD Biosciences | 1:250 | Surface |
| CCR7 | BUV395 | 150503 | 625526 | BD Biosciences | 1:160 | Surface |
| Live Dead UV Blue | UV Blue | - | L34962 | Thermo Fisher Scientific | 1:770 | Surface |
| CD4 | BUV496 | SK3 | 624283 | BD Biosciences | 1:320 | Intracellular |
| CD25 | BUV563 | 2A3 | 565699 | BD Biosciences | 1:320 | Surface |
| CD39 | BUV661 | TU66 | 624285 | BD Biosciences | 1:80 | Surface |
| CD95 | BUV737 | DX27 | 624286 | BD Biosciences | 1:80 | Surface |
| CD8 | BUV805 | SK1 | 612889 | BD Biosciences | 1:160 | Surface |
| IL-2 | BV421 | MQ1-17H12 | 564164 | BD Biosciences | 1:80 | Intracellular |
| CD154 | BV480 | TRAP1 | 624278 | BD Biosciences | 1:80 | Intracellular |
| CD45RO | BV570 | UCHL1 | 304226 | Biolegend | 1:320 | Surface |
| IL-17A | BV605 | BL168 | 512326 | Biolegend | 1:80 | Intracellular |
| Ki67 | BV650 | B56 | 563757 | BD Biosciences | 1:160 | Intracellular |
| CD69 | BV711 | FN50 | 563836 | BD Biosciences | 1:320 | Intracellular |
| TNF-α | BV750 | MAb11 | 566359 | BD Biosciences | 1:320 | Intracellular |
| CD27 | BV786 | L128 | 624292 | BD Biosciences | 1:320 | Surface |
